# A global survey of changing patterns of food allergy burden in children

**DOI:** 10.1186/1939-4551-6-21

**Published:** 2013-12-04

**Authors:** Susan L Prescott, Ruby Pawankar, Katrina J Allen, Dianne E Campbell, John KH Sinn, Alessandro Fiocchi, Motohiro Ebisawa, Hugh A Sampson, Kirsten Beyer, Bee-Wah Lee

**Affiliations:** 1 World Allergy Organization, Milwaukee, USA (headquarters; 2 International Inflammation (in-FLAME) Network, of the Worldwide Universities Network (WUN), Perth, Australia; 3 School of Paediatrics and Child Health, University of Western Australia, Perth, Australia; 4 Division of Allergy, Department of Pediatrics, Nippon Medical School, Tokyo, Japan; 5 Royal Children’s Hospital, Murdoch Children’s Research Institute and the University of Melbourne, Melbourne, Victoria, Australia; 6 Westmead Children’s Hospital and University of Sydney, Sydney, Australia; 7 Royal North Shore Hospital, Sydney and University of Sydney, Sydney, Australia; 8 Pediatric Hospital Bambino Gesù, Rome, Vatican City, Italy; 9 Clinical Research Center for Allergology and Rheumatology, Sagamihara National Hospital, Sagamihara, Kanagawa, Japan; 10 Icahn School of Medicine at Mount Sinai, New York, NY, USA; 11 Department of Pediatric Pneumology and Immunology, University Children’s Hospital Charité, Berlin, Germany; 12 Department of Paediatrics, Yong Loo Lin School of Medicine, National University of Singapore, Singapore, Singapore; 13 University of Western Australia, School of Paediatrics and Child Health Research, Princess Margaret Hospital for Children, Perth, WA, Australia

**Keywords:** Food allergy, Allergic disease, Allergy epidemic, Allergy prevention, Food allergens

## Abstract

While food allergies and eczema are among the most common chronic non-communicable diseases in children in many countries worldwide, quality data on the burden of these diseases is lacking, particularly in developing countries. This 2012 survey was performed to collect information on existing data on the global patterns and prevalence of food allergy by surveying all the national member societies of the World Allergy Organization, and some of their neighbouring countries. Data were collected from 89 countries, including published data, and changes in the health care burden of food allergy. More than half of the countries surveyed (52/89) did not have any data on food allergy prevalence. Only 10% (9/89) of countries had accurate food allergy prevalence data, based on oral food challenges (OFC). The remaining countries (23/89) had data largely based on parent-reporting of a food allergy diagnosis or symptoms, which is recognised to overestimate the prevalence of food allergy. Based on more accurate measures, the prevalence of clinical (OFC proven) food allergy in preschool children in developed countries is now as high as 10%. In large and rapidly emerging societies of Asia, such as China, where there are documented increases in food allergy, the prevalence of OFC-proven food allergy is now around 7% in pre-schoolers, comparable to the reported prevalence in European regions. While food allergy appears to be increasing in both developed and developing countries in the last 10–15 years, there is a lack of quality comparative data. This survey also highlights inequities in paediatric allergy services, availability of adrenaline auto-injectors and standardised National Anaphylaxis Action plans. In conclusion, there remains a need to gather more accurate data on the prevalence of food allergy in many developed and developing countries to better anticipate and address the rising community and health service burden of food allergy.

## Introduction

Food allergy has emerged as an unanticipated ‘second wave’ of the allergy epidemic [1], dramatically increasing the burden of allergic diseases in infants and preschool children [2,3]. In some highly industrialized regions the prevalence of food allergy in infancy has reached 10% [3] and there are now reports that rates of food allergy are now following the steep gradient of economic transition in rapidly developing countries [4]. While the rising global burden of asthma [5], rhinitis [6] and eczema [7] has been well-documented over the past 20 years through world-wide epidemiological research programs such as the ISAAC Study (International Study of Asthma and Allergies in Childhood), there have been, as yet, no equivalent published data on food allergies on a global platform. Although several centres collected data enriched for food allergy in association with more ISAAC recent surveys [8-10] global trends have not been well studied, likely because in the early 1990’s when ISAAC was established food allergy was relatively uncommon and more difficult to accurately ascertain than other allergic outcomes by questionnaire.

While there have been Systematic Reviews on food allergy prevalence, these largely capture data from Western Europe and North America where the majority of studies have been performed; many more than a decade ago [11]. The majority of these reports were based only on self-reported food reactions rather than objective measures of true IgE-mediated food allergy, and the few that used the gold standard, oral food challenges (OFC) did not have consistent or standardized criteria in defining outcomes [11]. New multi-centre well designed food allergy prevalence studies using OFC and IgE measures (notably the EuroPrevall birth cohort) are now underway and will more accurately determine the prevalence and cost food allergy [12]. The EuroPrevall study (more than 12,000 children) is also predominantly focused in Europe, although it has been extended east to examine an anticipated 37,000 children in the emerging economies of Russia, China, and India – countries comprising 40% of the global population and in transition from traditional to modern lifestyles [13]. However, the accurate determination of the food allergy burden in many other developing regions of the world remains an important unmet need.

The main purpose of this study was to utilize the global network of the World Allergy Organization (WAO) to provide a current ‘snapshot’ of the level of knowledge on the prevalence, patterns and burden of food allergens throughout the 93 national and regional member societies of WAO, and in particular reveal regions where more data is needed. We also sought to determine the level of service provision for food allergy, urban–rural preventionhealth-care access, availability of adrenaline auto injectors and standardized national food allergy anaphylaxis action plans in each country. This was also an opportunity to obtain some level of information about infant feeding practices in these diverse countries.

## Methods

This was a collaborative project between the WAO and the Worldwide Universities Network (WUN). The Survey targeted the 93 peak national and regional member societies of WAO, with instructions to be completed by the most qualified experts in paediatric food allergy in each country. A working group from the WUN International Inflammation (*inFLAME*) Network was established to develop the web-based questionnaire in February 2012. This included clinicians specialized in general paediatrics, paediatric allergy, neonatology, gastroenterology, nutrition and dietetics and epidemiology (each as members of both WAO and WUN). Questions were developed and discussed in a workshop format and then circulated to the wider *inFLAME* Network for comment. The resulting questionnaire was ratified by the WAO Special Committee on Food Allergy and Nutrition in June 2012, and disseminated through the WAO secretariat to the 93 regional/national societies in September 2012 (where possible data were also collected from neighboring non-WAO member countries). The respondents were identified by their own societies as the most qualified people to complete the questionnaire.

Each country was asked to provide any data to support any *change* in food allergy (both IgE and non-IgE forms as defined by WAO criteria [14] provided) focusing on the last 10 years, and provide the source and level of evidence that their answer was based on, including published research, national data, changing health-care burden or to indicate if there was insufficient data in their region to provide an answer. Regions reporting a change in prevalence were asked to indicate the age group most affected (infants <1 year; 1–5 year olds; or children <5 years) again with the nature and source of data that this was based on. All were asked if there was published information of any kind available regarding the overall prevalence of food allergy in their country, and to provide any data available on the estimated percentage of children in their population who have food allergy (all ages and for preschool [<5 years] and for school aged children [<5 years], if data available). They were also asked to indicate the most common clinical presentations of food allergy including (1) acute IgE-mediated food allergy (i.e. onset generally within 1–2 hours with angioedema, urticaria, vomiting or anaphylaxis); (2) non IgE-mediated food allergies (i.e. more delayed gastrointestinal symptoms only e.g. reflux, constipation, colic, chronic diarrhea, blood in stool, without IgE associated symptoms) or (3) mixed IgE and non-IgE food allergy (children with features of both acute onset symptoms and more chronic symptoms, such as eczema, exacerbated by foods). Each country representative respondent was asked to indicate the 5 most common food allergen triggers in order of prevalence for infants and children younger than 5 years, and a separate list for children older than 5 years. For each answer the respondents were asked to provide the source of data, if any. Information was also collected on the availability of adrenaline auto-injectors for treatment of anaphylaxis in each country (including if these are subsidized by the health care system and whether they are available widely or only through selected health services or to selected patient groups). The use of National Anaphylaxis Action Plans was also determined for each country (i.e. whether patients with a risk of anaphylaxis because of known allergy are given the same nationally standardized management plan). General information was also collected about the population size of each country and the numbers of trained paediatric allergists in the region. Finally, respondents were asked to provide information on early infant feeding and weaning practices including average duration of breastfeeding, age of introduction of complementary feeding foods, kinds of solid foods first used and whether there were any specific allergy prevention guidelines in relation to infant feeding. A six-month period was allowed for all responses to be received. Where there was more than one respondent from a country (i.e. more than one national allergy society, or multiple responses for any other reasons) the data was selected based on the highest level of evidence provided. In each case a literature search was performed by the investigators to confirm the cited data source, and to look for any additional evidence. Where data were also provided and/or published for neighboring non-WAO member counties this has also been included. The findings are presented for each country separately and arranged by geographical region.

## Results

**Figure 1:**
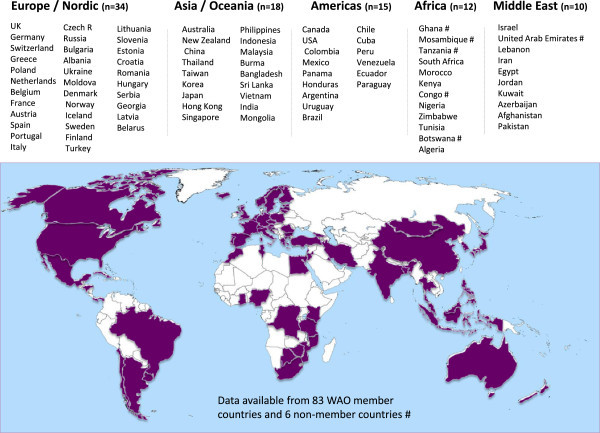
List and distribution of countries who participated in the survey or which had published data available on food allergy prevalence.

Data from 89 countries (Figure 1) were compiled to formulate a global picture of food allergy, including 12 countries in Western Europe, 5 Nordic countries and 17 in Central/Eastern Europe, 18 countries in Asia and Oceania, 15 countries in the Americas, 10 in the Middle East and 12 countries in Africa (Additional file 1: Table S1, on line repository). This included 83 WAO member countries and 6 non-member countries (Figure 1). These were categorized by the best level of evidence available for each country, with the highest level based in food allergy prevalence defined by oral food challenges (OFC) in unselected populations (Additional file 1: Table S1). The next best level of evidence was based on a suggestive clinical history confirmed by allergen skin prick testing (SPT) or other measures of food-specific IgE, also in unselected populations. Studies that relied only on self-reporting (such as questionnaire or phone survey data) were categorized as the lowest level of evidence for defining population food allergy prevalence.

### Food allergy prevalence by level of evidence

#### a). Food allergy defined by oral food challenge

**Figure 2:**
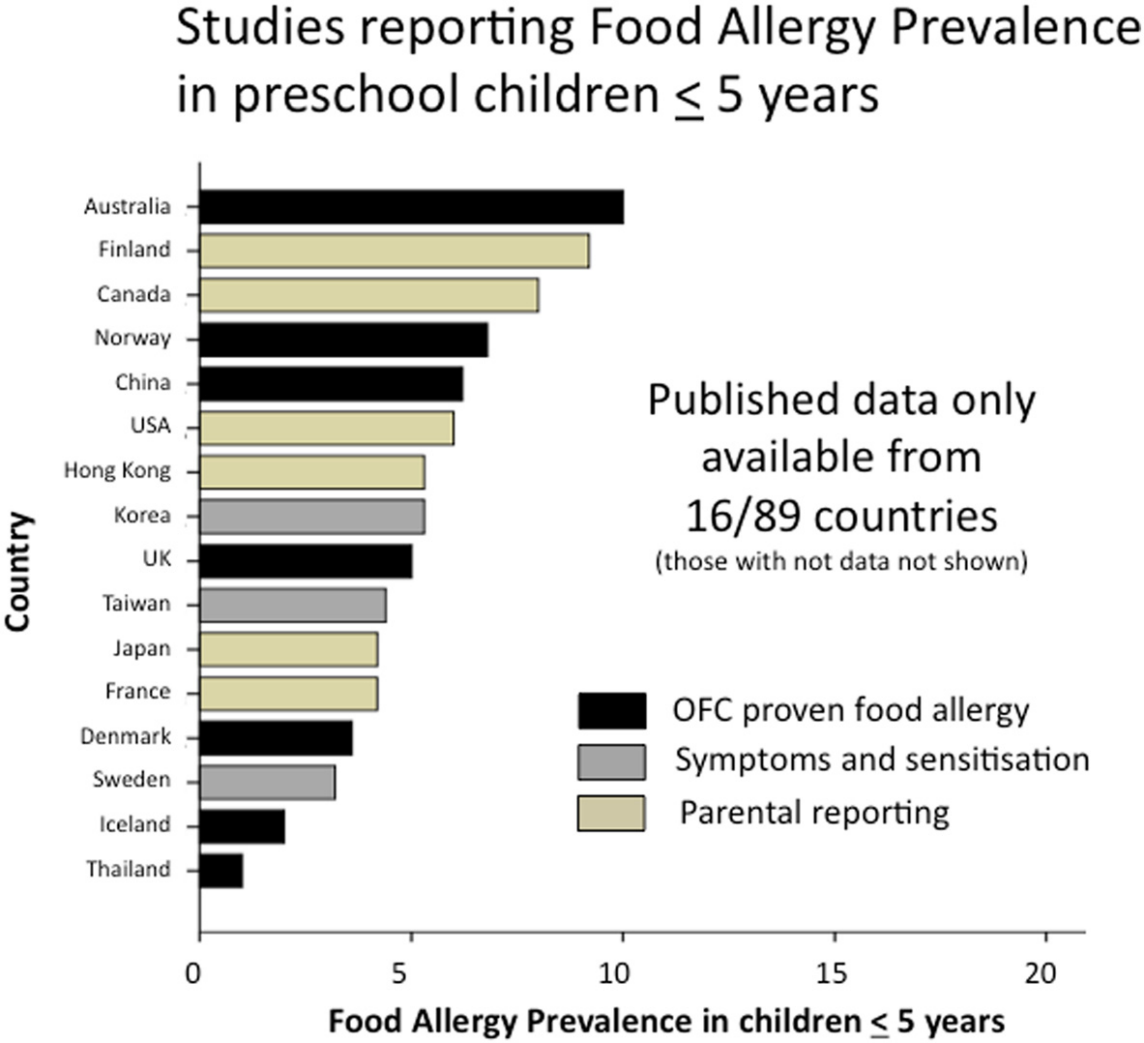
**Summary of food allergy prevalence from studies that provided data for children aged 5 years or less.** Studies are categorised according to level of evidence; OFC proven food allergy (black bars); food allergy based on symptoms and sensitisation (grey bars) or questionnaires/parental reporting (yellow bars).

**Figure 3:**
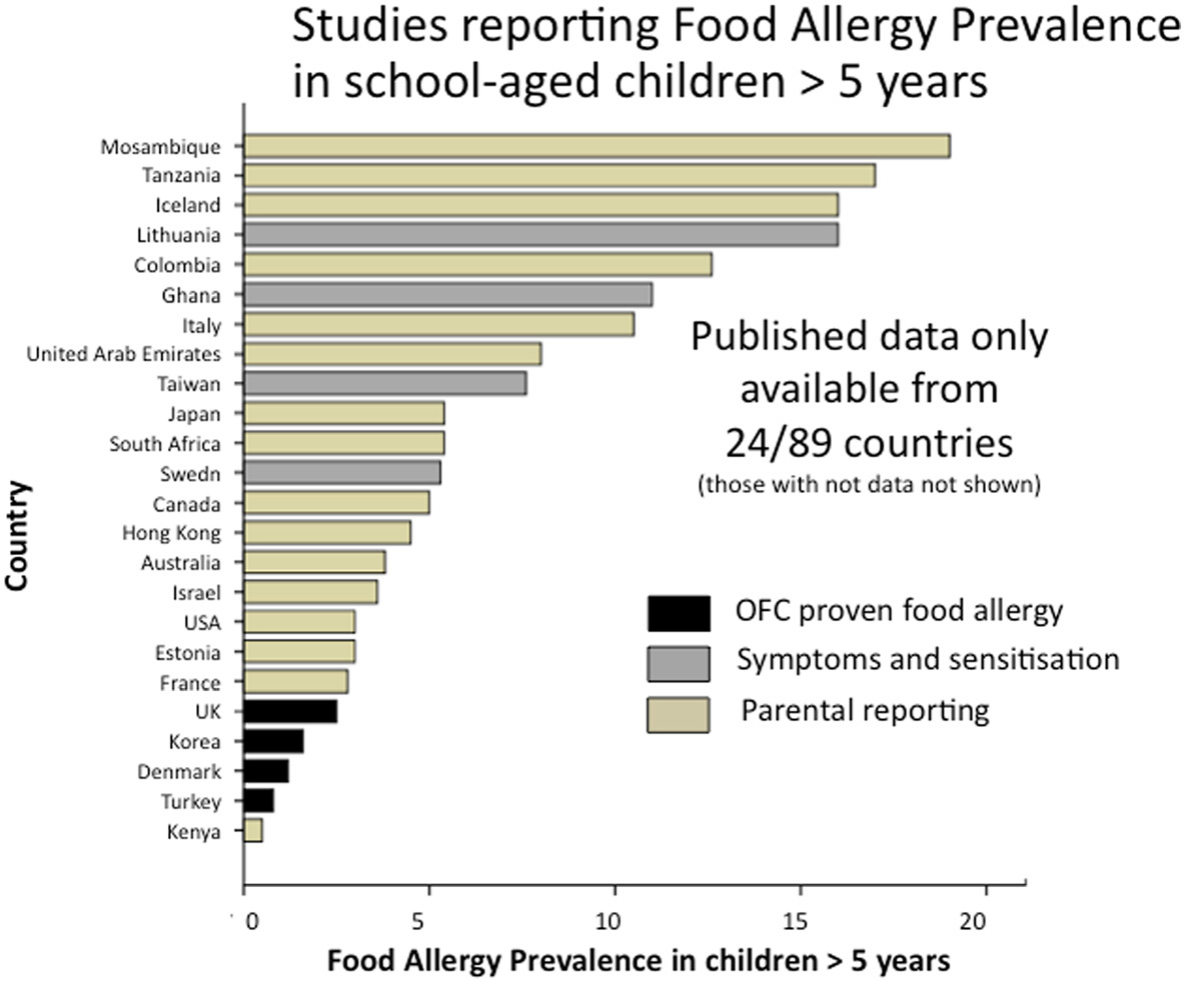
**Summary of food allergy prevalence from studies that provided data for children older than 5 years.** Studies are categorised according to level of evidence; OFC proven food allergy (black bars); food allergy based on symptoms and sensitisation (grey bars) or questionnaires/parental reporting (yellow bars).

**Figure 4:**
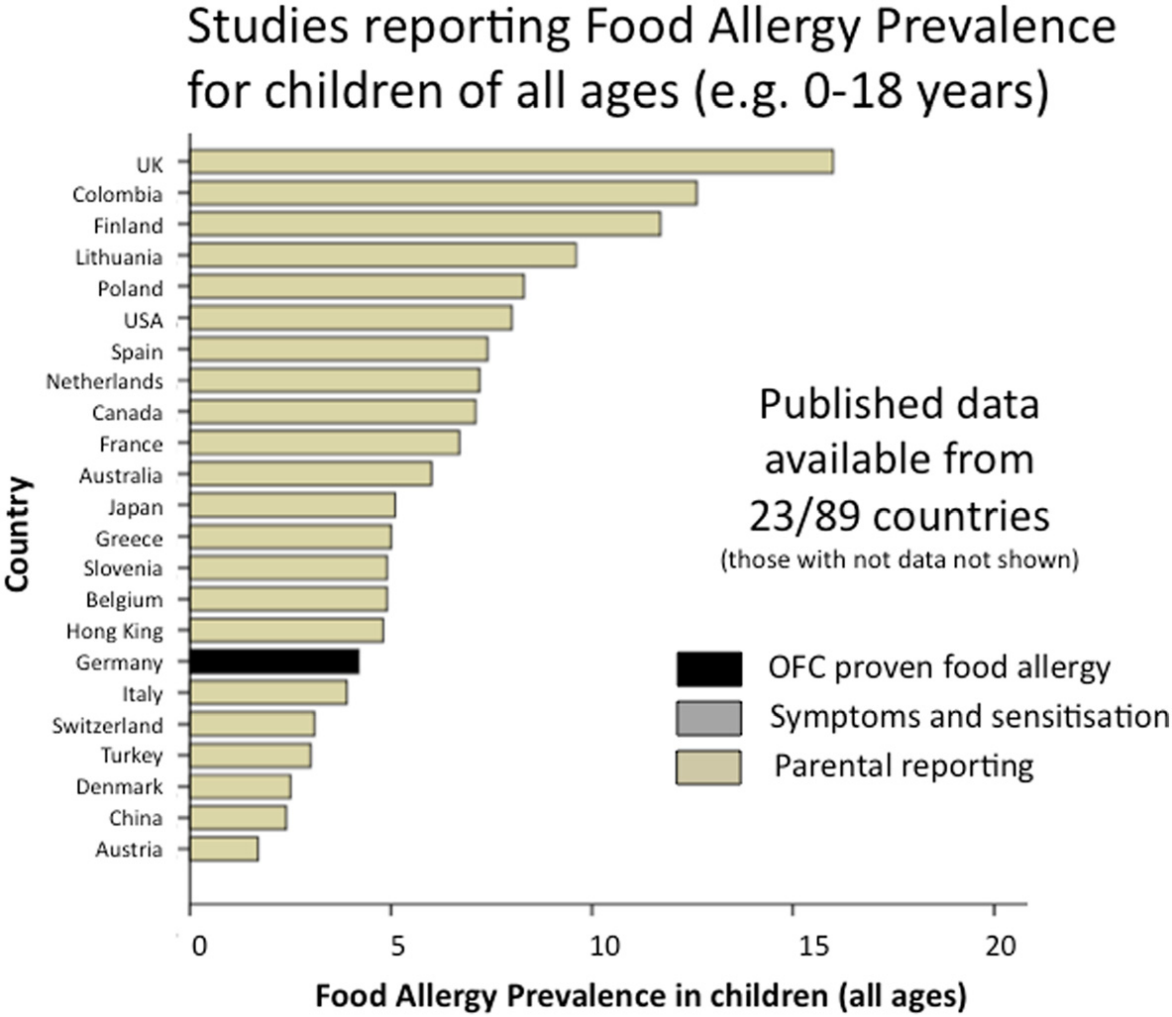
**Summary of food allergy prevalence from studies that provided data for children of all ages (generally ranging 0–18 years).** Studies are categorised according to level of evidence; OFC proven food allergy (black bars); or questionnaires/parental reporting (yellow bars).

Over one-half of the countries (51/89) had no food allergy prevalence data of any kind. Only 10% (9/89) of countries had accurate food allergy prevalence data based on OFC (Additional file 1: Table S1). In infants and preschool children (<5 years), food allergy prevalence based on OFC ranged from 1% in Thailand [15] to 10% in Australia [3] (Figure 2). Notably, new studies from south-western China show a comparable prevalence of OFC-proven food allergy in infants (3.8% [16], 6.2% [17], 7.7% [18]) as reported in European countries such as the UK (4%) [19], Denmark (3.6%) [20] and Norway (6.8%) [21]. The proportion of school-aged children (< 5 years) with OFC-proven food allergy was lower in all regions, ranging from <1% in Turkey (0.16%) [22] to 2.5% in the UK [23] although there were very few studies using OFC in this age group (Figure 3). In keeping with these findings a German study that studied 739 children across all ages (0–17 years, mean age 9.2 years) also found OFC-confirmed food allergy in 4.2%, with higher rates in younger children [24] (Figure 4).

#### b). Food allergy based on history of clinical reaction and demonstration of food-specific IgE

There have also been relatively few studies to determine food allergy prevalence based on a specific IgE testing in combination with a convincing clinical history, however these report food allergy rates in a similar range as the OFC-based studies [25-29]. In preschool children food allergy prevalence based on history and food-specific IgE was 3.2% in northern Europe (Sweden) [27] with very similar rates in Asia (3.4% in Taiwan) [26] (Figure 2). However, the prevalence in older children (based on symptoms and IgE testing) varied from only 0.3% (in Korea) [25] to 5.3% in Sweden [27] and 7.6% in Taiwan [26] (Figure 2). One of the very few African studies to investigate both food allergy symptoms and food specific IgE, in Ghanaian school children (5– 16 years), documented a high rates of reported food reactions (11%) and positive SPT (5%) [28]. However this is of uncertain significance as there was no clear association between reported adverse reactions to food and SPT or specific IgE levels [28]. Notably this rate of food-specific IgE is quite similar to other studies in Africa which revealed that 5.4% of unselected Xhosa high school students in Cape Town (n = 212) are SPT positive to foods [30], although clinical data were not available in the latter study.

#### c). Food allergy based on by questionnaire (self or parental reporting)

The majority of data currently available on food allergy prevalence are only based on self-reporting or parent-reporting questionnaire data (Additional file 1: Table S1). These generally yield much higher rates of food allergy than reports based on OFC or specific IgE-confirmed food allergy. In Canada [31] and the USA [32] survey reports suggest childhood food allergy prevalence rates of around 7-8%, with similar rates reported in the middle east (UAE) and some regions of Europe (Spain [33], Poland [34], the Netherlands [35] and France [36]). Even higher rates, around 15%, have been reported in other areas of western Europe such as the UK [12] and Germany [12], and in Iceland [12] and Colombia. Notably, self-reported life-time prevalence of food allergy in African countries such as Mosambique (n = 509) is as high as 19% [37] and self-reported food allergy from a survey of 400 households in Tanzania (ages not given) has been reported as high as 17% [38]. In South America (Colombia), a cross-sectional population survey (n = 3099) revealed 10% of 1–8 year olds and 12% of 9–16 year olds report food allergy [39], which is comparable to rates reported in Spain [12] and Italy [12]. On the other hand, lower rates of self/parent-reported food allergy have been published for countries in Asia, including Japan [40-42], Hong Kong [43] and Korea [44], where prevalence is reported to be around 5% or less. Similarly, the self/parent-reported prevalence of food allergy in some European countries, such as Slovenia [34], Estonia [8], Switzerland [34], Greece [34] and Belgium [34] has also been less than 5% in some studies. In Israel the prevalence of self-reported food allergy was also found to be, on average, less than 5% (3.2% in Jewish children and 5.0% in Arabic children) [10].

Evidence that food allergy rates are likely to be over-estimated come from countries where there are both OFC data and parent-reported food allergy. For example in a Turkish study of 3500 schoolchildren aged 6–9-years, parent-reported food allergy (5.7%) was significantly higher than the prevalence confirmed by OFC (0.80%) [45]. Similarly in Germany, parent-reported food reactions was 14.5% in baseline data from siblings of 1570 German infants enrolled in EuroPrevall [12] compared with 4.2% in a population study (n = 739) in which symptomatic children proceeded to allergy testing and OFC [24]. Inaccuracy of self/parent-reported food allergy is also evident in the wide variability between studies in the same country. For example, parent-reported food allergy was as high as 32% in 1513 Polish children enrolled in EuroPrevall [12] whereas in another study (telephone survey) of 8,825 children across 10 European regions only 8.3% of Polish children were reported to have food allergy [34].

#### d). Countries currently lacking any prevalence data

Regions of the world where food allergy prevalence data (of any kind) are particularly lacking are Central and South America, Africa, Eastern Europe and the Middle East.

### Food allergy prevalence trends by country/region

#### a) Time trends

The vast majority of countries reported an increase in food allergy prevalence in the preceding 10 years, although in most cases this was based on increasing health care burden (Additional file 1: Table S1). There was published evidence to support an increasing prevalence from Australia [2,46], Japan [41], China [18], Korea [47], USA [48] and Norway [21,49]. In the Chinese study, the same methodology was used in the same population 10 years apart and showed a doubling of challenge-proven IgE-mediated food allergy from 3.5% in 1999 to 7.7% in 2009 [18]. Another 39 countries reported an increase based on rising health care burden. Several countries provided published evidence of stable prevalence, including the UK [50], Finland [51,52] and Canada [53,54]. Relatively few countries (mostly in Europe) claimed stable prevalence based on health care service burden (rather than published data), including Greece, Austria, Romania, Georgia, the Czech Republic and Brazil. No country reported a declining burden of food allergy. For the remaining 35 countries there was insufficient data to determine (or even estimate) a change in prevalence.

In countries reporting an increase in food allergy, most (73%) reported that this was seen in children less than 5 years of age (Additional file 1: Table S1), although this was generally based on changing health care burden rather than published data. Of the 45 countries reporting an increase, 12 (27%) reported the greatest increase in infants < 1 year, 18 (40%) in 1–5 year olds, and only 2 (4.4%) in children older than 5 years. Of the remainder, 10 (22%) did not specify an age group and 3 countries (7%) indicated only that the increase was in children < 5 years (without specifying further).

#### b) Differences in prevalence between regions

Based on the most accurate measures (OFC) the highest reported prevalence of food allergy is in Australia. This was based on a large scale (n = 5,000) population–based cross-sectional study of food allergy in 12 month old infants in Melbourne, Australia [3]. Analysis of the first 2848 infants assessed indicated 10% have challenge-proven IgE-mediated food allergy (8.9% with egg allergy and 3.0% with peanut allergy) [3]. There were also very high rates of atopy with 18.0% showing food sensitization [3]. While this is higher than rates of challenge-proven food allergy reported in other westernized regions, such as Europe where rates are reportedly between 4-7% [19-21], it is notable in rapidly developing countries such as China the prevalence of challenge-proven food allergy is also approaching 8% in some studies [18]. It should be noted that different methods and different extracts were used to assess food sensitization. For example, some studies used native foods (such as raw egg [3]) where others used commercial extracts for skin prick testing.

At present there is very little quality data on food allergy from other regions in Asia, or from South America or from Africa where there are unexpectedly high rates of food sensitisation [28,30].

#### c) Common Food Allergens by region

The most common food allergens in children less than 5 years of age were relatively similar across all regions, generally including cows milk, egg, peanuts and seafood, with regional variations in the relative frequency of these (Additional file 1: Table S2). In Oceania (Australia [3] and New Zealand) and Asia [16,40,44,55] egg allergy appears to be more common than milk allergy, whereas the reverse is true in the Americas [31,32,39,56] and the Middle east, cows milk allergy is consistently reported as more common. In Europe the pattern is more variable but both egg and cows milk tend to be the two most common allergens in this age group. In Asia seafood (fish and shellfish) are also consistently in the ‘five most common food allergens’ in preschool children, with some unusual allergens reported, such as ant eggs [15].

In older children (<5 years) there is slightly more diversity in most common food allergens reported, but peanuts, tree nuts, seafood, egg and milk tend to be common to most regions. In many European countries, fruit allergens such as apple and kiwi feature consistently as common allergens [34] and this was also seen in Central and South American countries [39,56] (Additional file 1: Table S2). Peanut and other nuts tend to be among the most common allergens reported in Australia, Western Europe and the USA. In Eastern Europe egg remains the most common allergen in a number of counties at this age [34,45,57,58]. In the Middle East sesame is frequently among the five most common allergens [10]. Beef allergy is among the most common in Turkish children both <5 years [59] and < 5 years of [45,57,59]. The few other regions that report allergies to meats among the five most common allergens include Poland [34], Colombia [39], and Mozambique [37], although data are limited.

#### d) Types of food allergy by region

The patterns of food allergy presentations also varied by region (Additional file 1: Table S2). Acute IgE-mediated symptoms are generally the most common form of food allergy, particularly in North America, Western Europe, Australia and the Middle East. Although this is also true in some regions of Asia (China, Japan, Hong Kong and Singapore) others report mixed presentation with patients more commonly showing both non-IgE and IgE-mediated features (Korea, Thailand, Phillipines, Malaysia, Indonesia and Bangladesh). Similarly in Eastern Europe non-IgE mediated symptoms (Belarus, Bulgaria) or mixed non-IgE and IgE-mediated symptoms (Lithuania, Slovenia, Estonia, Croatia and Latvia) are more common. Mixed features are also more typical in South Africa, Ghana and Mozambique. In South and Central America some countries report IgE symptoms as the most common (Argentina, Cuba, Brazil and Mexico) whereas other report mixed features (Colombia and Honduras) or non-IgE mediated presentations (Uruguay and Chile) as the most common.

### Infant feeding patterns by region

#### a) Introduction of complementary feeding

The reported age and type of complementary food introduction, based upon reported common practice, not official recommendations, is shown in Additional file 1: Table S2. In the significant majority of countries (35/42, 83%) it was reported that complementary feeding is most commonly started between 4–6 months, including both developed and developing regions of South America, Eastern Europe, South Africa, and across the whole Asian region. The main exceptions were in the Middle East where ‘solid’ foods are reportedly introduced even earlier at 3–4 months in some regions (Egypt, Jordan and Kuwait). Only Austria and Uruguay reported commonly introducing solid foods after 6 months of age.

The first complementary foods in most regions included rice cereals, fruits and vegetables with regional differences reflecting dietary variations (Additional file 1: Table S2). Rice cereal is among the most common first foods in Australia, North America and some areas of Europe. Rice and fruits are also common first foods in Asia. Vegetables and fruits are the most common starting foods in many European countries. Legumes, rice and vegetables are reported to be the first foods in the Middle Eastern regions.

As anticipated, parents are reported to be commonly concerned about trying to prevent allergic disease in the more westernized regions such as North America (and Mexico), Western Europe, Oceania (Australia and New Zealand), Nordic regions and some regions in Asia (Japan and Korea). In other regions of Asia, South and Central America, Africa and the Middle East, experts report that parental concern about allergy is reported to be generally less common or rare.

#### b) Infant formula usage

We did not attempt to gather accurate data on breastfeeding patterns as this is either published elsewhere or not available in some regions. However we did seek to obtain some information on the use of infant formulas, although this was based on clinician practices and experiences rather than published data (Additional file 1: Table S2). While this information is crude, the trends do suggest frequent early (perinatal) use of formulas (i.e. prior to hospital discharge) in a number of regions, and use by parents in the first 4 months. In particular, relatively high rates of complementary feeding in the first 4 months were reported in South Africa, Eastern Europe (Bulgaria, Czech Republic, Croatia and Georgia), Brazil, Mexico and Israel. Lower rates of early formula use were noted in other Middle Eastern countries, Nordic countries, Asia, Oceania and the UK.

### Paediatric allergy services and anaphylaxis services by country/region

Each regional society was asked to provide the number of registered trained paediatric allergists (or equivalent) in their country, and based on the population size, that was used to determine the number of specialist service providers per million of total population (Additional file 1: Table S3). There were wide variations in the number of service providers, with some countries (such as the UK) with a reported high burden of food allergy having < 1 paediatric allergist per million of population (0.32/million). Similarly, other high-burden countries such as Australia, New Zealand and Canada, report ≤ 3 paediatric allergists per million population. The USA and European regions with relatively high food allergy burden are generally better serviced (such as Germany, Greece, Italy and Nordic countries) but this is highly variable. Other regions where food allergy is emerging as a new and growing problem have very few trained specialists per head of population. This is true in Asia (with the exception of Japan), South America (with the exception of Cuba) and the Middle East (with the exception of Israel), where there are generally ≤ 3 paediatric allergists per million of population. African nations have very few trained paediatric specialist, although there is a general lack of data on both the burden of disease and service provision in that region. In the most populous regions (China and India), there were also no accurate data available.

Many countries reported perceived urban–rural differences in paediatric allergy health care services (Additional file 1: Table S3). The main exceptions are smaller developed countries or states (such as Singapore, Hong Kong and smaller European regions) or regions where services are equally poor in urban and rural regions (such as Mongolia).

### Adrenaline autoinjectors and allergy action plans

There are wide variations in the availability of adrenaline auto-injectors and many countries do not yet have standardized national Food Allergy/Anaphylaxis Action Plans (Additional file 1: Table S3). Adrenaline auto-injectors are readily available in countries with the higher service burden of allergy, such as North America, Western Europe, Nordic countries and Oceania, and are generally subsidized by healthcare systems. Most, but not all, of these countries have standardized national anaphylaxis action plans. However, in some countries with a very high service burden of allergy (such as in New Zealand) adrenaline auto-injectors are still not subsidized by the healthcare system. There is a reported lack of general availability of adrenaline auto-injectors (very difficult to access and/or not subsidized) in the Middle East, many areas of Asia and South America, Africa (although data more limited) and some regions of Eastern Europe.

## Discussion

The main purpose of this survey was to provide a global ‘snap shot’ of current trends in food allergy including the quality of evidence available and regions where more information is needed. Because the rise in food allergy is believed to have been relatively recent as ‘a second wave’ of allergy after the rise in asthma and rhinitis [1,60], most regions do not have accurate or current prevalence data, particularly in infants and young children under 5 years of age who are most commonly affected. Many estimates are based on parent- or self-reported questionnaires or surveys and very few objectively confirm the prevalence of food allergy through the gold standard of oral food challenge. Even those few studies undertaking food challenges until recently have been hampered by poor challenge participation rates resulting in potential for substantial allergic bias.

The findings reveal a general paucity of quality data on food allergy prevalence, even in high prevalence areas of North America and Western Europe. In Europe this will be addressed to a large extent by the large-scale multicentre ‘Europrevall Study’. Extending this to study an additional 37,000 children in the emerging economies of Russia, China, and India [13] will be very helpful. However, there remains a need for similar studies in South America, South East Asia, the Middle East and Africa where very little information is available. To address this in South Africa there are plans for a cross-sectional, observational study of IgE-mediated food allergy in an unselected population of children aged 12–36 months. This will include both urban and rural communities and all children with a positive SPT of any size (≥1 mm) will be referred to the Allergy clinic for OFC to determine the clinical significance. Similar studies are also needed in other emerging economies.

A recent meta-analysis commissioned by the National Institute of Allergy and Infectious Disease concluded that the prevalence of food allergy across all age groups is likely to be between 1% and 10%, with higher prevalence in younger age groups [61]. The more recent well designed Australian HealthNuts study supports this, with 10% of 1 year old infants demonstrating challenge-proven IgE-mediated food allergy with even higher rates of food sensitisation [3]. The design of HealthNuts provides a good model for future food allergy prevalence studies. The cohort was not selected on the basis of a family history of atopy, and children with positive tests irrespective of SPT wheal size proceeded to hospital-based food challenge to confirm the clinical food allergy. Oral food challenges were the first to be undertaken with predetermined objective stopping criteria. Prevalence was adjusted for participation bias, both at the point of initial population screening and at invitation for food challenge, and nonparticipants were also surveyed to allow for any bias towards allergy. This study was clearly labor-intensive and it is acknowledged that, although OFC are the gold standard in diagnosing food allergy, this is not feasible in many low and middle-income countries.

Another key observation is that rapidly developing regions such as China are showing significant rates of food allergy [17,18]. Thus, despite common notions that food allergy is less prevalent in Asia, these prevalence figures from various studies in Asia are comparable to those reported from western populations (Additional file 1: Table S1). Published studies on food allergy time-trends confirm the rising prevalence in Asia [18], and a gradient of food allergy with progressive economic development also provides indirect evidence that food allergy is associated with westernisation; with higher rates of food allergy in Chinese born in Hong Kong (4.8%) compared with those born in mainland China (2.4%) [4]. This is consistent with recent reports showing a rise in non-food allergic diseases such as asthma and allergic rhinitis in developing countries that are adopting a more “westernised” lifestyle [62-65]. Eczema prevalence has also increased significantly in Asia [7]. This has major global implications, as the heavily populated regions of Asia are becoming rapidly urbanised, westernised and industrialised.

In this context it is important to note preliminary evidence that Asian and other non-Caucasian populations may be even more susceptible to the adverse effects of ‘Westernisation’ than Caucasians [66,67]. Earlier studies of respiratory disease observed that both allergic symptoms and sensitisation were more common in Asian Australians than non-Asians Australians [66]. Rates were also higher in Australian-born Asians than Asian immigrants, with the prevalence increasing with length of stay in Australia [66] and this was echoed recently in data from the Healthnuts study on eczema prevalence. More recent US studies have similarly noted that non-white races are more susceptible to food allergy, particularly Asian populations [67], suggesting a strong genetic propensity that is amplified by a western environment. Thus, as urbanisation inevitably progresses we can anticipate a major rise in the global burden of food allergy.

Many countries (n = 45) already report an increase in food allergy prevalence, although only 6 based this on published evidence and the remainder based this on reported increased health care burden. Only 9 countries reported a stable prevalence, and none reported a decline in food allergy burden. However, many (n = 35) could/did not provide any data (either published or based on health care burden). We did not collect data on the changing prevalence of specific allergies, however, it has been recently noted that while peanut allergy has increased dramatically in the USA [68], UK [69,70] and Australia [46], this is less evident in Asia although data are more limited (reviewed recently in [55]).

Although there is some variation in the patterns of food allergy, there is surprising consistency in the most common allergen triggers, particularly in infants and younger children. In most regions egg and milk are among the most common allergens in preschool children, generally listed as the two most common. Although these are typically considered as ‘transient’ allergies, it is interesting that these were also noted among the most common allergens in older children (<5 years) in almost all regions. It is not clear how this might relate to emerging evidence that egg and milk allergies are now commonly persisting into late childhood and adolescence [71,72]. Peanut, tree nuts and seafood were also common allergen triggers in almost all regions for both preschool and older children. Across continental Europe, fruits appear to be more common allergens than in the UK, Australia and North America, particularly in older children and this is likely related to tree pollen cross sensitisation. In Asia, seafood including shrimp and shellfish feature commonly, even in preschool children. There was a similar trend in South America although there is less data to support this. Sesame was more frequently listed among common allergens in the Middle East.

Although it was beyond the scope of this survey to examine the relationship between early feeding patterns and food allergy, we collected very basic information on the patterns of early infant formula use, the age at which infants most commonly start complementary foods, and the level of parental concern about allergy prevention in each region. As noted above, there were regional differences in the likelihood that complementary formula milk are used in the perinatal period (but hospital staff prior to discharge) and in the early postnatal period by parents, however there was general uniformity in the age at which other complementary foods are most commonly introduced (typically reported to be between 4–6 months in most regions). As anticipated, parental concern about allergy prevention was more common in the high prevalence regions, but is an emerging issue in some developing regions. We acknowledge that this is qualitative data, based on the experience and knowledge of experts in each region, rather than more accurate population-level census data, and should be interpreted as such.

The burden of food allergy and eczema also has major economic implications for health care provision of specialist allergy services worldwide. Our survey reveals wide inequities in health service provision, even in developed regions where the disease burden is already well recognised. Based on current trends in emerging economies the health burden of food allergy and eczema is anticipated to rise substantially in the next decade, and the greatest impact is likely to be in the more populated regions of the developing world. Currently, emerging economies generally have the fewest trained medical staff with expertise in paediatric allergy. Investment in training, which can take many years, is particularly important as these regions might anticipate a substantial increase in disease burden.

There were also wide variations in the reported access to emergency treatments for potentially life threatening food allergy (anaphylaxis). Experts from many emerging economies report that adrenaline auto-injectors are not readily available. In other countries, cost is reported to be a significant barrier because of lack of government subsidies, even in some highly industrialised countries where a high burden of food allergy is recognised. Many countries still do not have nationally-standardised Food Allergy/Anaphylaxis Action plans, even where auto-injectors are available, with the choice of plan remaining at the discretion of each individual treating clinician. This has implications for the consistency and training for teachers and other community carers supervising children with food allergies. These observations generally suggest the need for greater awareness among governments and health policy makers of the significance and impact of food allergy particularly in children.

## Conclusions

In summary, this survey highlights food allergy as a significant paediatric health issue that is likely to increase globally in the coming decade. In some developed economies 1 in 10 infants now have challenge-proven IgE-mediated food allergy, following substantive increase in the last 10 years [2,46]. Similar trends are now apparent in the developing countries of Asia and other regions. The survey also reveals the paucity of quality data in many regions, and the need to obtain more accurate information about food allergy prevalence and impact, even in developed countries. Unlike asthma, food allergy has been relatively neglected area of allergy research. This is important to address this as part of promoting awareness of food allergy for health policy and health care system to better anticipate the growing impact and growing need for better services, community education and training to cope with this rising global health issue.

## Competing interests

The authors declare that they have no competing interests.

## Authors’ contributions

SP coordinated the study, chaired questionnaire design workshops. She also analysed the data and drafted the manuscript. RP provided genera advice for the study, logistic support and advice on the questionnaires and data analysis. KA, DC, JS and SP all assisted with the questionnaire development and manuscript content and review. AF, ME, HS KB and BL reviewed the process and the manuscript. All authors read and approved the final manuscript.

## Supplementary Material

Additional file 1 **Table S1.** Food allergy prevalence by region. **Table S2.** Food allergy patterns and feeding practices by region. **Table S3.** Food allergy health services (in 2012). Click here for file
