# Supplementary material for: A global survey of changing patterns of food allergy burden in children
Source: World Allergy Organ J. 2013 Dec 4;6(1):21. doi: 10.1186/1939-4551-6-21 (PMC3879010; doi:10.1186/1939-4551-6-21)
Supplement: Additional file 1 — Table S1. Food allergy prevalence by region. Table S2. Food allergy patterns and feeding practices by region. Table S3. Food allergy health services (in 2012). [file 1939-4551-6-21-S1.docx]

**Table S1: FOOD ALLERGY PREVALENCE BY REGION**

| Unselected population: FA confirmed by food challenges | Unselected population: FA confirmed by sensitization | Unselected population: questionnaire / self-reporting only | Selected population study (descriptive data) | No data |
| --- | --- | --- | --- | --- |

1. **OCEANIA and ASIA PACIFIC**

| **Region** | **Country** | **Prevalence of clinical food allergy in last 10 years (%)** | | | **Method of determining prevalence, population size (and reference to support response if available)** | **Change in prevalence in last 10 years?** | **Age group most affected by any change?** |
| --- | --- | --- | --- | --- | --- | --- | --- |
|  |  | **All ages** | **< 5 year olds** | **> 5 year olds** |  |  |  |
| **Oceania** | Australia | (6%) | 10%[^1^](#_ENREF_1) | 3.8% (nuts only) [^2^](#_ENREF_2) | [^1^](#_ENREF_1)Population-based sample of 2,848 infants at 1 year using predetermined **SPT and OFC** criteria to measure outcomes. Other percentages (in brackets) estimates only based on other data sets. | Increased [^3^](#_ENREF_3)^,^ [^4^](#_ENREF_4) | < 5years [^3-5^](#_ENREF_3) |
|  | New Zealand | - | - | - | [^6^](#_ENREF_6)Recognized lack of data ** | Increased* | < 5years * |
|  |  |  |  |  |  |  |  |
| **Asia** | China | 2.4[^7^](#_ENREF_7) | 3.8%[^8^](#_ENREF_8) 6.2%[^9^](#_ENREF_9)  7.7% [^10^](#_ENREF_10) | - | [^8^](#_ENREF_8) 497 consecutive infants attending well-baby checks. Food allergy history**, SPT and OFC.**  [^9^](#_ENREF_9) Study across 3 cities, based on food allergy history**, SPT and OFC** (0-2 year olds).  [^10^](#_ENREF_10) Same methodology 10 years apart showed increase from 3.5% 1999 to 7.7% in 2009 in in Chongqing, China  [^7^](#_ENREF_7) Indirect evidence of increasing food allergy comparing higher rates of food allergy in Chinese born in Hong Kong (4.8%) versus Mainland China (2.4%). | Increased [^7^](#_ENREF_7) [^10^](#_ENREF_10) | - |
|  | Thailand | - | 1%[^11^](#_ENREF_11)  0.4%[^12^](#_ENREF_12) | - | [^11^](#_ENREF_11)Survey to parents of 546 children (2-7 years), 9.3% reported FA and underwent **SPT and OFC** (1% positive)  [^12^](#_ENREF_12) Survey to parents of 656 children (6 months-6 years) and 6.2% reported FA and underwent **SPT and OFC** (0.45% positive) | Increased* | < 1year * |
|  | Taiwan | - | 3.4% [^13^](#_ENREF_13) | 7.6% [^13^](#_ENREF_13) | [^13^](#_ENREF_13) Nationwide, cross-sectional, random questionnaire-based survey of 30,018 people. Outcome based on Convincing history +/- SPT/IgE | - | - |
|  | Korea | - | 5.3%[^14^](#_ENREF_14) | 0.6% (6-7 year olds) [^15^](#_ENREF_15) 1.6% (12-13 year olds) [^15^](#_ENREF_15) | [^15^](#_ENREF_15) Population-based questionnaire FA survey of 6-7 year olds (n=3907) and 12-13 year olds (n=3975) with specific IgE in children with positive responses.  [^14^](#_ENREF_14) Birth cohort (n=1177) convincing history of immediate allergic reactions to food in first 12 months. | Increased[^16^](#_ENREF_16) | > 5 years [^16^](#_ENREF_16)  (practice burden < 1 year*) |
|  | Japan | 5.1%[^17^](#_ENREF_17) | 4.2%[^18^](#_ENREF_18)  5.1%*  9.2%** | 3%- 5.4% [^18^](#_ENREF_18)  3.1%*  1.3%** | [^18^](#_ENREF_18)^,^ [^19^](#_ENREF_19) Questionnaire survey to parents of 14,669 Japanese schoolchildren (90% response) aged 7–15 years. Rate of food avoidance based on history of reactivity to foods. 4.2% avoidance in infancy, 5.4% at 7 years and 3% at 15 years.  [^17^](#_ENREF_17) Larger Japanese survey (0-6yr) also based on food avoidance (n=101,322) [^20^](#_ENREF_20)  * Study in Japanese (personal communication Motohiro Ebisawa) population study Sagamihara city enrolled 5932 infants at 4 mo, 5.1% (of 2888) followed to 3 years, and 3.1% (of 2165) followed to 7 years had history of diagnosed food allergy.  ** Study in Japanese (personal communication Motohiro Ebisawa) based on rate of food elimination in survey of 101,322 children from 892 nursery schools. 9.2% at 1 year and 1.3% at 6 years were avoiding one or more food for suspected food allergy. | Increased [^18^](#_ENREF_18) | - |
|  | Hong Kong | 4.8%[^21^](#_ENREF_21) | 4.6% [^7^](#_ENREF_7) -  5.3%[^21^](#_ENREF_21) | 4.5%[^21^](#_ENREF_21) | [^21^](#_ENREF_21) Population based survey of 7,393 children aged 0-14 years. History of convincing adverse reactions to foods.  [^7^](#_ENREF_7) Community survey of 3827 preschool children (2–7 yr), parent-reported reactions (8.1%) and parent-reported doctor diagnosed reactions (4.6%) | Increased* | 1-5 years* |
|  | Singapore | - | 1.2% (shellfish);  0.7% [^22^](#_ENREF_22) (nuts) | 0.3% [^23^](#_ENREF_23) (fish);  5.2% (shellfish);  0.54% [^22^](#_ENREF_22) (nuts) | No data found on overall FA prevalence.  [^22^](#_ENREF_22) Nut and shellfish allergy prevalence in 4-6 year olds (n=4390 and 14-16 year olds (n=6450) participating in regional survey  [^23^](#_ENREF_23) Fish allergy prevalence in the same population study. | Increased* | 1-5 years* |
|  | Philippines | - | - | 2.3% [^23^](#_ENREF_23) (fish);  5.1% (shellfish);  0.7% [^22^](#_ENREF_22) (nuts) | No data found on overall FA prevalence.  [^23^](#_ENREF_23) Fish allergy prevalence in 11,434 14-16 year olds participating in regional survey  [^22^](#_ENREF_22) Nut and shellfish allergy prevalence in the same population study | Increased* | 1-5 years* |
|  | Indonesia | - | - | - | **No population prevalence data reported/found | Increased * | < 1year * |
|  | Malaysia | - | - | - | **No population prevalence data reported/found | Increased * | < 1year * |
|  | Burma | - | - | - | **No population prevalence data reported/found | - | - |
|  | Bangladesh |  |  |  | **No population prevalence data reported/found | Increased * | >5year * |
|  | Sri Lanka | - | - | - | **No population prevalence data reported/found | - | - |
|  | Vietnam | - | - | - | **No population prevalence data reported/found | - | - |
|  | India | - | - | - | **No population prevalence data reported/found | - | - |
|  | Mongolia |  |  |  | **No population prevalence data reported/found | Increased * | >5year * |

1. **AMERICAS**

| **Region** | **Country** | **Prevalence of clinical food allergy in last 10 years (%)** | | | **Method of determining prevalence, population size (and reference to support response if available)** | **Change in prevalence in last 10 years?** | **Age group most affected by any change?** |
| --- | --- | --- | --- | --- | --- | --- | --- |
|  |  | **All ages** | **< 5 year olds** | **> 5 year olds** |  |  |  |
| **North America** | Canada | 7.1%[^24^](#_ENREF_24) | (8%) | (5%) | [^24^](#_ENREF_24)Self reporting telephone interviews in 10 provinces (9,667 individuals including 2,198 children). Other percentages (in brackets) estimates only based on other data sets. | Stable [^25^](#_ENREF_25)^,^ [^26^](#_ENREF_26) | - |
|  | USA | 3.4%[^27^](#_ENREF_27) – 8%^[28](#_ENREF_28)^ | (6%) | (3%) | [^27^](#_ENREF_27)Physician diagnosis review of electronic medical records (9,184 low-income, minority children 0-21 years).  [^28^](#_ENREF_28)Randomized, cross-sectional electronic survey (40,104 children 0-18 years).  Other percentages (in brackets) estimates only based on other data sets. | Increased[^29^](#_ENREF_29) | < 5years [^30^](#_ENREF_30) |
|  |  |  |  |  |  |  |  |
| **Central and South America** | Colombia | 10.1-12.6% [^31^](#_ENREF_31) |  | 12.6% [^31^](#_ENREF_31) | [^31^](#_ENREF_31)Cross-sectional population survey  of 3099 (aged 1-83 years) in Cartagena. 10% self reporting of food allergy for 1-8 year olds, 12% for 9-16 year olds | - | - |
|  | Mexico | 3%[^32^](#_ENREF_32) | - | - | [^32^](#_ENREF_32) Selected population attending allergy clinics. No population data available. | Increasing* | 1-5 years olds* |
|  | Panama | - | - | - | **No population prevalence data reported/found | - | - |
|  | Honduras | - | - | - | **No population prevalence data reported/found | Increased * | < 1years * |
|  | Argentina | - | - | - | **No population prevalence data reported/found | Increased * | < 1years * |
|  | Uruguay | - | - | - | **No population prevalence data reported/found | Increased * | < 1years * |
|  | Brazil | - | - | - | **No population prevalence data reported/found | Stable | - |
|  | Chile | - | - | - | **No population prevalence data reported/found | Increased * | < 1years * |
|  | Cuba | - | - | - | **No population prevalence data reported/found | Increased * | > 5years * |
|  | Peru | - | - | - | **No population prevalence data reported/found | - | - |
|  | Venezuela | - | - | - | **No population prevalence data reported/found | - | - |
|  | Ecuador | - | - | - | **No population prevalence data reported/found | - | - |
|  | Paraguay | - | - | - | **No population prevalence data reported/found | - | - |

**3) EUROPE**

| **Region** | **Country** | **Prevalence of clinical food allergy in last 10 years (%)** | | | **Method of determining prevalence, population size (and reference to support response if available)**  **Change in prevalence in last 10 years?**  **Age group most affected by any change?** | | |
| --- | --- | --- | --- | --- | --- | --- | --- |
|  |  | **All ages** | **< 5 year olds** | **> 5 year olds** |  |  |  |
| **Western Europe** | United Kingdom | 16%[^33^](#_ENREF_33) | 4-6%[^34^](#_ENREF_34) | 2.5% ^[35](#_ENREF_35)^ | [^34^](#_ENREF_34) Both parent reporting and medical assessments with **SPT and OFCs** (in birth cohort of 969 assessed a 1 year [^34^](#_ENREF_34), 3years [^36^](#_ENREF_36) and 6 years [^35^](#_ENREF_35))  [^33^](#_ENREF_33) Parent-reported food hypersensitivity (from baseline data from siblings of 1140 UK infants enrolled in EuroPrevall). | Stable [^37^](#_ENREF_37) | - |
|  | Germany | 4.2[^38^](#_ENREF_38) -14.5%[^33^](#_ENREF_33) | - | - | [^33^](#_ENREF_33) Parent-reported food hypersensitivity (from baseline data from siblings of 1570 German infants enrolled in EuroPrevall)  ^[38](#_ENREF_38)^ Standardized telephone parent interviews of 739 children (0-17yrs) followed by examination, **SPT and OFC** in symptomatic individuals. | Increased* | 1-5 years* |
|  | Switzerland | 3.1% [^39^](#_ENREF_39) | - | - | [^39^](#_ENREF_39) Telephone survey – parental reporting on 8,825 children across 10 European regions | Increased* | 1-5 years* |
|  | Greece | 4.8[^39^](#_ENREF_39) -5.1% [^33^](#_ENREF_33) | - | - | [^33^](#_ENREF_33) Parent-reported food hypersensitivity (from baseline data from siblings of 1080 Greek infants enrolled in EuroPrevall).  [^39^](#_ENREF_39)Telephone survey – parental reporting on 8,825 children across 10 European regions. | Stable* | - |
|  | Poland | 8.3[^39^](#_ENREF_39) – 32%[^33^](#_ENREF_33) | - | - | [^33^](#_ENREF_33) As above - in 1513 Polish children enrolled in EuroPrevall). [^39^](#_ENREF_39) As above. | Increased* | 1-5 years* |
|  | Netherlands | 7.2[^40^](#_ENREF_40) -25.6%[^33^](#_ENREF_33) | - | - | [^40^](#_ENREF_40) Questionnaire - parent reported food hypersensitivity in 4450 children ( 4-15yrs). Data >10 years old.  [^33^](#_ENREF_33) As above - in 976 Dutch children enrolled in EuroPrevall). | Increased* | 1-5 years* |
|  | Belgium | 4.9[^39^](#_ENREF_39) | - | - | [^39^](#_ENREF_39) As above. | Increased* | Infants < 1 year* |
|  | France | 6.7%[^41^](#_ENREF_41) | 4.19%[^42^](#_ENREF_42) | 2.80%[^42^](#_ENREF_42) | [^42^](#_ENREF_42) Population based questionnaire to 44,000 people (adults and children) 75% response rate. Second questionnaire to 1,129 people who has suggestive symptoms.  [^41^](#_ENREF_41) Questionnaire to 3500 school-aged children (parent reporting 77.6% response rate) | - | - |
|  | Austria | 1.7[^39^](#_ENREF_39) | - | - | [^39^](#_ENREF_39) As above. | Stable* | - |
|  | Spain | 7.4%[^43^](#_ENREF_43) -9.8%[^33^](#_ENREF_33) |  |  | [^33^](#_ENREF_33) EuroPrevall as above: Parent-reported food hypersensitivity (from baseline data from siblings of 1387 Spanish infants).  [^43^](#_ENREF_43) Not a population study: Selected population of 4991 new patients prospectively recruited in allergy clinics. Included adults. | Increased* | 1-5 years* |
|  | Italy | 3.9[^39^](#_ENREF_39) - 10.2%[^33^](#_ENREF_33) | - | 10.5%[^44^](#_ENREF_44) | [^33^](#_ENREF_33) EuroPrevall as above: Parent-reported food hypersensitivity (from baseline data from siblings of 1486 Italian infants).  [^39^](#_ENREF_39) As above.  [^44^](#_ENREF_44) Parental reporting, questionnaire in 900 children 5-14 years (69% response rate) | Increased* | - |
|  | Portugal | - | - | - | **No population prevalence data reported/found | Increased* | Infants < 1 year* |
|  |  |  |  |  |  |  |  |
| **Central/**  **Eastern Europe** | Turkey | 3%  (data from national meeting; no reference provided) | - | 0.16%[^45^](#_ENREF_45),  0.80%^(a)^ -  5.7%^(b)^ [^46^](#_ENREF_46) | [^45^](#_ENREF_45) From 6963 adolescents in ISAAC Phase II, those with symptoms and **positive IgE underwent OFC**  ^[46](#_ENREF_46)^ Parental-reported IgE-mediated food allergy ^(b)^ (prevalence 5.7%) then confirmed by **DBPCFC** ^(a)^ (prevalence 0.80%) in 3500 schoolchildren aged 6–9-years. | Increased* | 1-5 years* |
|  | Lithuania | 9.6%[^33^](#_ENREF_33) | - | 12.8% [^47^](#_ENREF_47) - 16% [^48^](#_ENREF_48) | [^33^](#_ENREF_33) EuroPrevall as above: Parent-reported food hypersensitivity (from baseline data from siblings of 1556 Lithuanian infants).  [^47^](#_ENREF_47) Questionnaire-survey of 540 schoolchildren.  [^48^](#_ENREF_48) Questionnaire- EuroPrevall survey of 3084 schoolchildren with specific IgE confirmation in symptomatic children | Increased* | >5 year olds* |
|  | Slovenia | 4.9%[^39^](#_ENREF_39) |  |  | [^39^](#_ENREF_39) As above. | - | - |
|  | Estonia | - | - | 3%[^49^](#_ENREF_49) | [^49^](#_ENREF_49) Parent self-reporting of food allergy ISAAC Phase II; 11-12 year olds in Sweden and Estonia. | - | - |
|  | Croatia |  |  |  | **No population prevalence data reported/found | Increased* | Infants < 1 year* |
|  | Romania | - | - | - | **No population prevalence data reported/found | Stable* | - |
|  | Hungary | - | - | - | **No population prevalence data reported/found | - | - |
|  | Serbia | - | - | - | **No population prevalence data reported/found | - | - |
|  | Georgia | - | - | - | **No population prevalence data reported/found | Stable* | - |
|  | Latvia | - | - | - | **No population prevalence data reported/found | Increased* | 1-5 years* |
|  | Belarus | - | - | - | **No population prevalence data reported/found | - | - |
|  | Czech R | - | - | - | **No population prevalence data reported/found | Stable* | - |
|  | Russia | - | - | - | **No population prevalence data reported/found | - | - |
|  | Bulgaria | - | - | - | **No population prevalence data reported/found | Increased* | >5 year olds* |
|  | Albania | - | - | - | **No population prevalence data reported/found | - | - |
|  | Ukraine | - | - | - | **No population prevalence data reported/found | - | - |
|  | Moldova | - | - | - | **No population prevalence data reported/found | - | - |
| **Nordic regions** | Denmark | 2.5[^39^](#_ENREF_39) | 1.2% -3.6%[^50^](#_ENREF_50)  2.3%[^51^](#_ENREF_51) | 1.2%[^52^](#_ENREF_52)  1% [^51^](#_ENREF_51) | [^39^](#_ENREF_39) As above.  [^50^](#_ENREF_50)DARC Cohort of 562 children, questionnaires, **specific IgE and OFC (milk, egg, and peanut).**  [^52^](#_ENREF_52) DARC Cohort at 6 years.  [^51^](#_ENREF_51) Cohorts of children <3 yr (n=111), 3 yr (n= 486 children), >3 yr (n=301) for questionnaires, SPT, specific IgE and OFC to the most common allergenic foods. | - | - |
|  | Norway | - | 6.8% ^[53](#_ENREF_53)^ | - | [^53^](#_ENREF_53) Birth cohort 512 children followed to 2.years, food allergy defined by history, **SPT and DBPCFC**.  [^54^](#_ENREF_54) Previous prevalence assessed in birth cohort of 2803 (parental reporting of reactions to foods (egg, fish, or nuts) and egg allergy confirmed by **DBPCFC** or IgE at 2.5yrs. | Increased [^53^](#_ENREF_53)^,^ [^54^](#_ENREF_54) | 1-5 years |
|  | Iceland |  | 2% [^55^](#_ENREF_55) | 16.2% [^33^](#_ENREF_33) | [^55^](#_ENREF_55) Prospective (n=1,341) birth cohort, children with possible food allergy had SPT and **double-blind OFC**. Second cohort (n=3000) also under analysis (published in Islandic)  [^33^](#_ENREF_33) EuroPrevall as above: Parent-reported food hypersensitivity (from baseline data from siblings of 1341 Icelandic infants). | Increased | 1-5 years |
|  | Sweden | - | 3.2%[^56^](#_ENREF_56) -11%[^56^](#_ENREF_56) | 5.3%[^56^](#_ENREF_56) | [^56^](#_ENREF_56) Questionnaires on 3694 children (90%) of the BAMSE birth cohort at 4 years. IgE measurements on 2563. Total perceived prevalence of food hypersensitivity reported (IgE and non-IgE) as 11%. Diagnosed disease with sensitisation 3.2% at 4yr [^56^](#_ENREF_56), 5.3% at 8 yrs [^56^](#_ENREF_56) | Increased* | 1-5 years* |
|  | Finland | 11.7%[^39^](#_ENREF_39) | 9.2 %[^57^](#_ENREF_57) | - | [^39^](#_ENREF_39) As above.  [^57^](#_ENREF_57) Parent reporting questionnaire of preschool history at school entry for 1542 Finnish children | Stable [^58^](#_ENREF_58)^,^ [^59^](#_ENREF_59) | - |

**4) MIDDLE EAST and AFRICA**

| **Region** | **Country** | **Prevalence of clinical food allergy in last 10 years (%)** | | | **Method of determining prevalence, population size (and reference to support response if available)** | **Change in prevalence in last 10 years?** | **Age group most affected by any change?** |
| --- | --- | --- | --- | --- | --- | --- | --- |
|  |  | **All ages** | **< 5 year olds** | **> 5 year olds** |  |  |  |
|  |  |  |  |  |  |  |  |
| **Middle East** | Israel | - | - | 3.6% [^60^](#_ENREF_60)  (3.2% in Jews; 5.0% in Arabs) | [^60^](#_ENREF_60) Self-report questionnaire on FA administered with the ISAAC study of 13–14 year olds (n=11,171) including 8757 Jews and 2414 Arabs. | Increased * | < 1year * |
|  | United Arab Emirates # | - | - | 8%[^61^](#_ENREF_61) | [^61^](#_ENREF_61) Multistage random population sample of 397 school children whose parents completed a self-administered questionnaire for history of physician diagnosis of FA | - | - |
|  | Lebanon | - | - | - | **No population prevalence data available.  [^62^](#_ENREF_62) Studies in selected patients with serum collected for suspected FA symptoms revealed 21% has specific IgE to foods) | Increased * | < 1year * |
|  | Iran | - | - | - | **No population prevalence data reported/found  Studies of food sensitisation in children with eczema (50%)[^63^](#_ENREF_63) and asthma (15%)[^64^](#_ENREF_64) provide some information on patterns of food sensitisation in Iran | - | - |
|  | Egypt |  |  |  | **No population prevalence data reported/found | Increased * | 1-5 years * |
|  | Jordan | - | - | - | **No population prevalence data reported/found | Increased * | 1-5 years * |
|  | Kuwait | - | - | - | **No population prevalence data reported/found | Increased * | >5years * |
|  | Azerbaijan | - | - | - | **No population prevalence data reported/found | - | - |
|  | Afghanistan | - | - | - | **No population prevalence data reported/found | - | - |
|  | Pakistan | - | - | - | **No population prevalence data reported/found | - | - |
|  |  |  |  |  |  |  |  |
| **Africa** | Ghana # |  |  | 11% food reactions and 5% SPT+ ^[65](#_ENREF_65)^ | [^65^](#_ENREF_65) Children (5– 16 years; n = 1,714) from 9 Ghanaian schools. Parental questionnaires on FA and atopy assessed by **SPT and specific IgE.** Poor correlation between SPT/specific IgE and symptoms. |  |  |
|  | Mosam-bique # | - | - | 19% (all ages) [^66^](#_ENREF_66) | [^66^](#_ENREF_66) Self-reported life-time history of food allergy (in 509 adults). Most commonly to seafood, meat, fruits and vegetables | - | - |
|  | Tanzania # | - | - | 17% [^67^](#_ENREF_67) | Self-reported food allergy (in 400 households). No information about ages or food triggers [^67^](#_ENREF_67) | - | - |
|  | South Africa | - | - | 5.4% [^68^](#_ENREF_68) sensitised to food | *No population FA prevalence data available.  ^[68](#_ENREF_68)^ A study in 212 unselected Xhosa high school students in Cape Town. SPT only, not based on clinical food allergy, most common SPT to egg white (3.3%), peanuts (1.9%) and milk (1.9%).  [^69^](#_ENREF_69) In selected populations (with eczema) there are high rates of sensitisation to egg (475); cow’s milk (28%) and peanut (27)%. | Increased * | 1-5 years * |
|  | Morocco | 2.5% study population show specific IgE to foods [^70^](#_ENREF_70) | - |  | **No population prevalence data reported/found  200 patients (adults and children) screened for sIgE to aeroallergens and foods (milk, egg, soy, wheat, peanuts) – reviewed in[^70^](#_ENREF_70) | - | - |
|  | Kenya | - | - | 0.5% (all ages) | **No population prevalence data reported/found  [^71^](#_ENREF_71) A 1992 study in children and adults (n=574) in general practice. FA defined by questionnaire only  [^72^](#_ENREF_72) A 1994 study of SPT in 72 adults showed sensitisation to egg, milk cabbage and fish | - | - |
|  | Congo# |  | - | - | **No population prevalence data reported/found  [^73^](#_ENREF_73)Selected population (n=423) patients with allergic rhinitis (children and adults) also tested to foods (crab, wheat, soy). 5% food sensitisation to foods in this selected population | - | - |
|  | Nigeria # | - | - | - | **No population prevalence data reported/found  [^74^](#_ENREF_74) Selected population of 1019 patient with eczema (mean age 13.8 years, range 1-59 years): 4% triggered by foods – egg, cray fish, milks). | - | - |
|  | Zimbabwe | - | - | - | **No population prevalence data reported/found.  [^75^](#_ENREF_75)Specific IgE testing to allergens in selected population showed high rates of sensitisation to foods (including Apple, tomato, crab, soy, peanut) | - | - |
|  | Tunisia | - | - | - | **No population prevalence data reported/found  [^70^](#_ENREF_70) Selected population (n=100) patients with eczema (children and adults) and 38% positive to egg. | - | - |
|  | Botswana# | - | - |  | **No population prevalence data reported/found  [^70^](#_ENREF_70) Selected population (n=64) allergic (children and adults). 14% with food allergy in this selected population (egg, peanut, milk). | - |  |
|  | Algeria | - | - | - | **No population prevalence data reported/found | - | - |

# Indicates: a country that does *not* have a WAO member society

**Table S2: FOOD ALLERGY PATTERNS AND FEEDING PRACTICES BY REGION**

**1) OCEANIA and ASIA PACIFIC**

| **Region** | **Country** | **Most common food triggers < 5 years** | | **Most common symptoms** (IgE, non-IgE, or mixed features) | **First weaning foods** (other than formula milk) | **Age of starting comple-mentary foods** (actual rather than recommended) | **Estimated perinatal use of formulas** (by hospital or nursery staff) | **Estimated use of any formulas**  **< 4 months**  (excluding the neonatal period) | **Is allergy prevention a common parental concern?** |
| --- | --- | --- | --- | --- | --- | --- | --- | --- | --- |
|  |  | **< 5 years** | **> 5 years** |  |  |  |  |  |  |
|  |  |  |  |  |  |  |  |  |  |
| **Oceania** | Australia | Egg[^1^](#_ENREF_1), peanut[^1^](#_ENREF_1), cows milk[^1^](#_ENREF_1), tree nuts and  soy* | peanut, tree nuts, egg, cows milk, seafood [^4^](#_ENREF_4)^,^ [^5^](#_ENREF_5) | IgE mediated | Rice cereal, pureed cooked vegetables, fruits, meat and fish | 4-6 months | <25% | 25-50% | Often |
|  | New Zealand | As above – no data | As above – no data | IgE mediated | As above | 4-6 months | <25% | 25-50% | Often |
|  |  |  |  |  |  |  |  |  |  |
| **Asia** | China | egg[^8^](#_ENREF_8), cows milk[^8^](#_ENREF_8), peanut[^8^](#_ENREF_8), fish[^8^](#_ENREF_8), shrimp[^8^](#_ENREF_8), and fruit[^8^](#_ENREF_8) | Shellfish[^9^](#_ENREF_9), peanut[^9^](#_ENREF_9), Egg[^9^](#_ENREF_9), cow milk[^9^](#_ENREF_9), fish[^9^](#_ENREF_9) | IgE mediated |  |  | - | - | - |
|  | Thailand | Cows milk[^11^](#_ENREF_11) shrimp[^11^](#_ENREF_11), hen eggs[^11^](#_ENREF_11), fish[^11^](#_ENREF_11), crab [^11^](#_ENREF_11), ant eggs[^11^](#_ENREF_11), wheat[^11^](#_ENREF_11) | Shrimp[^12^](#_ENREF_12), (crab, other shellfish and wheat) | Non-IgE features | Rice, banana, orange, cereals, vegetables | 4-6 months | 25-50% | 25-50% | Sometimes |
|  | Taiwan | Milk[^13^](#_ENREF_13), shrimp[^13^](#_ENREF_13), fish[^13^](#_ENREF_13), crab[^13^](#_ENREF_13), peanut[^13^](#_ENREF_13) | Shrimp[^13^](#_ENREF_13), Crab[^13^](#_ENREF_13), Fish[^13^](#_ENREF_13), Mango[^13^](#_ENREF_13), Mollusc[^13^](#_ENREF_13), Milk[^13^](#_ENREF_13),  Peanut[^13^](#_ENREF_13) | - | - | **-** | - | - | - |
|  | Japan | Egg[^17^](#_ENREF_17), Cows milk[^17^](#_ENREF_17), wheat[^17^](#_ENREF_17), peanut[^17^](#_ENREF_17), fish, fruits | Egg[^18^](#_ENREF_18), cows milk[^18^](#_ENREF_18), wheat[^18^](#_ENREF_18), shellfish[^18^](#_ENREF_18), fruits and peanut[^18^](#_ENREF_18) | IgE mediated | Rice, potatoes, vegetables, fruits, soy paste. | 4-6 months | 50-75% | 25-50% | Often |
|  | Korea | Egg[^14^](#_ENREF_14), cows milk[^14^](#_ENREF_14), peanut[^14^](#_ENREF_14), wheat[^14^](#_ENREF_14), (soy and fish*) | Egg (6-7 years)[^15^](#_ENREF_15), shellfish and fruits (12-13years)[^15^](#_ENREF_15), (nuts and grains*) | Mixed (IgE and non-IgE features) | Rice soup, fruits, vegetables, beef, beans | 4-6 months | 25-50% | 50-75% | Often |
|  | Hong Kong | Shellfish [^7^](#_ENREF_7)^,^ [^21^](#_ENREF_21), egg[^21^](#_ENREF_21), peanut [^7^](#_ENREF_7), milk[^21^](#_ENREF_21), fish[^8^](#_ENREF_8), fruits[^21^](#_ENREF_21) | - | IgE mediated | - | - | - | - | - |
|  | Singapore | [^20^](#_ENREF_20)Egg, milk, peanut, tree nut, shellfish | Egg, Peanut, tree nut, shellfish,  fish[^23^](#_ENREF_23) [^22^](#_ENREF_22), cow milk | IgE mediated | Rice, fruits, vegetables, fish, tubers | 4-6 months | < 25% | 25-50% | Rarely |
|  | Philippines | Seafood, eggs, milk soy, peanut (no data) | Shellfish,  Peanut, fish[^23^](#_ENREF_23) [^22^](#_ENREF_22) (egg, cow milk, soy) | Mixed (IgE and non-IgE features) | Rice, banana, vegetables, orange, cereals, fish, meat | 4-6 months | < 25% | 25-50% | Rarely |
|  | Indonesia | Cows milk and egg (no data) | Seafood, eggs, nuts | Mixed IgE and non-IgE symptoms | Fruit, biscuit, porridge (wheat or rice) vegetables, meat | 4-6 months | < 25% | 25-50% | Rarely |
|  | Malaysia | Cows milk, egg, peanut, tree nuts, sea food (no data) | Sea food, chicken, peanut, egg | Mixed IgE and non-IgE symptoms | Potatoes, sweat potato, rice, carrot, fruits | 4-6 months | 50-75% | 25-50% | Sometimes |
|  | Burma | (no data) | - |  |  |  | - | - | - |
|  | Bangladesh | (no data) | - | Mixed IgE and non-IgE symptoms | - | 4-6 months | < 25% | < 25% | Sometimes |
|  | Sri Lanka | (no data) | - | - | - | - | - | - | - |
|  | Vietnam | (no data) | - |  |  |  | - | - | - |
|  | Vietnam | (no data) | - |  |  |  | - | - | - |
|  | India | (no data) | - | - | - | - | < 25% | < 25% | Rarely |
|  | Mongolia | (no data) | - | - | - | - | - | - | - |

**2) AMERICAS**

| **Region** | **Country** | **Most common food triggers < 5 years** | | **Most common symptoms** (IgE, non-IgE, or mixed features) | **First weaning foods** (other than formula milk) | **Age of starting comple-mentary foods** (actual rather than recommended) | **Estimated perinatal use of formulas** (by hospital or nursery staff) | **Estimated use of any formulas**  **< 4 months**  (excluding the neonatal period) | **Is allergy prevention a common parental concern?** |
| --- | --- | --- | --- | --- | --- | --- | --- | --- | --- |
|  |  | **< 5 years** | **> 5 years** |  |  |  |  |  |  |
|  |  |  |  |  |  |  |  |  |  |
| **North America** | Canada | Cows milk[^24^](#_ENREF_24) Egg, peanut[^24^](#_ENREF_24) tree nuts, fish and soy[^24^](#_ENREF_24) | Peanut, tree nuts, cows milk, egg, seafood, wheat[^24^](#_ENREF_24) | IgE mediated | Rice cereal, other cereal grains, bananas, other fruits, pureed vegetables | 4-6 months | <25% | 25-50% | Often |
|  | USA | Cows milk[^28^](#_ENREF_28), peanut[^27^](#_ENREF_27), eggs[^27^](#_ENREF_27)^,^ [^28^](#_ENREF_28), shellfish, tree nuts, fish (soy) | Peanuts[^27^](#_ENREF_27)^,^ [^28^](#_ENREF_28), shellfish[^27^](#_ENREF_27)^,^ [^28^](#_ENREF_28), milk[^28^](#_ENREF_28), tree nuts[^27^](#_ENREF_27)^,^ [^28^](#_ENREF_28), egg [^27^](#_ENREF_27)^,^ [^28^](#_ENREF_28) | IgE mediated | Rice cereal, oat cereal, carrot, sweet potatoes other vegetables | 4-6 months | 25-50% | >75% | Often |
|  |  |  |  |  |  |  |  |  |  |
| **Central and South America** | Colombia | Cows milk, egg, sea food, vegetables (limited data) [^31^](#_ENREF_31) | Fruits and vegetables[^31^](#_ENREF_31), seafood, meats, milk, egg (limited data). [^31^](#_ENREF_31) | Mixed IgE and non-IgE symptoms | - | - | - | - | - |
|  | Mexico | Cows milk[^32^](#_ENREF_32), egg[^32^](#_ENREF_32), fish, shrimp, soy (wheat) | Egg, milk, wheat, soy, fish (others: beans, chili, mango,cacao, strawberry[^32^](#_ENREF_32)) | IgE mediated | Apple, pear, banana, potato, carrot | 4-6 months | >75% | >75% | Often |
|  | Panama | - | - | - | - | - | - | - | - |
|  | Honduras | Cows milk, soy, egg, wheat, fruits (no data) | Cows milk, soy, egg, wheat, fruits, peanuts (no data) | Mixed IgE and non-IgE symptoms | Apple, banana, potato, sweet potato, pear | 3-4 months | 25-50% | 25-50% | Rarely |
|  | Argentina | Cows milk, egg, wheat, peanut, soy (no data) | Cows milk, peanut, tree nuts, shellfish, fish (no data) | IgE mediated | Potatoes, carrots, apple, banana, pumpkin | 4-6 months | 25-50% | <25% | Rarely |
|  | Uruguay | Cows milk, egg, soy, wheat, shrimp, (no data) | Cows milk, egg, wheat, soy, shrimp, fruits (no data) | Non-IgE features | Carrots, potatoes, banana, apple, chicken | >6 months | < 25% | 25-50% | Rarely |
|  | Brazil | Cows milk, egg, soy, wheat, corn, (no data) | Fish, shellfish, tree nuts, peanuts, eggs (no data) | IgE mediated | Apple, orange, banana, cereals, egg yolk | 4-6 months | > 75% | 25-50% | Sometimes |
|  | Chile | Cows milk, egg, soy, legumes, corn, vegetables (no data) | Egg, soy, wheat, fruits, vegetables (no data) | Non-IgE features | Potatoes, pumpkin, chard, beef, apple | 4-6 months | 25-50% | < 25% | Sometimes |
|  | Cuba | Cows milk, egg, wheat, soy, fish (no data) | Egg, cows milk, peanut, soy, wheat (no data) | IgE mediated | Fruits, vegetables, cereals, meat, egg yolk | 4-6 months | - | - | Sometimes |
|  | Peru | (no data) | - | - | - | - | - | - | - |
|  | Venezuela | (no data) | - | - | - | - | - | - | - |
|  | Ecuador | (no data) | - | - | - | - | - | - | - |
|  | Paraguay | (no data) | - | - | - | - | - | - | - |

**3) EUROPE**

| **Region** | **Country** | **Most common food triggers < 5 years** | | **Most common symptoms** (IgE, non-IgE, or mixed features) | **First weaning foods** (other than formula milk) | **Age of starting comple-mentary foods** (actual rather than recommended) | **Estimated perinatal use of formulas** (by hospital or nursery staff) | **Estimated use of any formulas**  **< 4 months**  (excluding the neonatal period) | **Is allergy prevention a common parental concern?** |
| --- | --- | --- | --- | --- | --- | --- | --- | --- | --- |
|  |  | **< 5 years** | **> 5 years** |  |  |  |  |  |  |
|  |  |  |  |  |  |  |  |  |  |
| **Western Europe**  **Western Europe** | United Kingdom | Egg, Cows milk, peanut, tree nuts, fish [^36^](#_ENREF_36) | Peanut, tree nut, cows milk, egg, fish (clinical observation) | IgE mediated | Rice, carrot, sweet potato, potato, apple and pear | 4-6 months | <25% | >75% | Often |
|  | Germany | Egg[^76^](#_ENREF_76), cows milk [^76^](#_ENREF_76), peanut [^76^](#_ENREF_76), wheat, soy, fish [^76^](#_ENREF_76) | Peanut [^76^](#_ENREF_76), wheat [^76^](#_ENREF_76), tree nuts[^38^](#_ENREF_38), apple and fruits[^39^](#_ENREF_39), vegetables [^38^](#_ENREF_38) | Mixed IgE and non-IgE mediated symptoms | Vegetables, fruit, cereal, meat products, dairy products, egg, and fish[^77^](#_ENREF_77) | 4-6 months | - | - | Often |
|  | Switzerland | Cows milk[^39^](#_ENREF_39), egg, wheat, peanut, hazelnut, potato[^78^](#_ENREF_78) | Milk [^39^](#_ENREF_39), Peanut, egg, fish, hazelnut, kiwi [^78^](#_ENREF_78) | IgE mediated | Apple, carrot, potatoes, pear | 4-6 months | <25% | 50-75% | Often |
|  | Greece | Egg, milk, fruits, meats, fish, legumes[^39^](#_ENREF_39) | Nuts, Egg, shellfish, milk, legumes, fruits | IgE mediated | Potato, apple, pear, rice, chicken, cereals | 4-6 months | 25-50% | 25-50% | Sometimes |
|  | Poland | Cows milk [^39^](#_ENREF_39) egg, wheat, peanut, soy | Cows milk, egg, fruits, meats, peanut, tree nuts, wheat [^39^](#_ENREF_39) | IgE mediated | - | - | - | - | - |
|  | Netherlands | Milk, egg, peanuts, tree nuts (clinical observation) | Peanuts, tree nuts, apple, pear, kiwi (no data source provided) | IgE mediated | - | - | - | - | - |
|  | Belgium | Cows milk, egg, soy, wheat | Milk, fruits, eggs, legumes, wheat, nuts, vegetables, fish [^39^](#_ENREF_39) | Mixed IgE and non-IgE mediated symptoms | - | - | - | - | - |
|  | France | Cow milk, eggs, kiwi, peanuts fish, tree nuts, and shrimp [^41^](#_ENREF_41) | Cow milk, eggs, kiwi, peanuts, fish, tree nuts, and shrimp [^41^](#_ENREF_41) | IgE mediated | - | - | - | - | - |
|  | Austria | Cows milk, egg, wheat, peanuts, fish | Fruits[^39^](#_ENREF_39), milk, wheat, vegetables, egg, nuts | - | Carrot, parsnip, potato, rice | > 6 months | < 25% | 25-50% | Often |
|  | Spain | Cows milk, egg, fish[^43^](#_ENREF_43) | Fruits, nuts, shellfish, egg,, milk, fish, legumes, vegetables and cereals [^43^](#_ENREF_43) | Mixed IgE and non-IgE symptoms | Fruits, rice and cereals, fish [^43^](#_ENREF_43) | - | - | - | - |
|  | Italy | Milk, eggs, wheat | Milk, fruits, wheat, eggs, fish, nuts, vegetables, fish [^39^](#_ENREF_39) | Mixed IgE and non-IgE symptoms | - | - | - | - | - |
|  | Portugal | Milk, egg, fish, nuts, cereals (based on clinical practice) | Egg, milk, fish, nuts, fruits (based on clinical practice) | IgE mediated | Cereals (rice, gluten free), vegetables, fruits, meat, wheat | - | 25-50% | 50-75% | Sometimes |
|  |  |  |  |  |  |  |  | | |
| **Central/**  **Eastern Europe** | Turkey | Cows milk, egg, wheat, beef, peanut[^79^](#_ENREF_79) | Egg, beef, tree nuts, cocoa, cows milk, kiwi, fish[^46^](#_ENREF_46)^,^ [^80^](#_ENREF_80) | IgE mediated | Cereals (including wheat), yogurts, fruits and vegetables, eggs | 4-6 months | < 25% | < 25% | Sometimes |
|  | Lithuania | Milk, egg, wheat, peanut[^81^](#_ENREF_81) (potato and fish) [^81^](#_ENREF_81) | Egg, cows milk, fruits [^47^](#_ENREF_47) | Mixed IgE and non-IgE symptoms | - | - | - | - | - |
|  | Slovenia | Egg, Cows milk, wheat[^39^](#_ENREF_39) | Egg, Cows milk, fruits, wheat, soy, nuts [^39^](#_ENREF_39) | Mixed IgE and non-IgE symptoms | - | - | - | - | - |
|  | Estonia | Egg, milk | Fruits, milk, egg, fish, wheat, nuts[^49^](#_ENREF_49) | Mixed IgE and non-IgE symptoms | - | - | - | - | - |
|  | Croatia | Egg, Cows milk, peanuts, wheat, soy (based on clinical practice) | Peanuts, fiah, soy, chicken, potato (based on clinical practice) | Mixed IgE and non-IgE symptoms | Apple, pear, banana, carrot, potato | 4-6 months | >75% | 25-50% | Sometimes |
|  | Romania | Cows milk, egg, wheat, chicken, carrot (based on clinical practice) | Egg, meats wheat, nuts, fruits (based on clinical practice) | IgE mediated | Apple, potato, carrots, wheat, chicken | 4-6 months | 25-50% | 25-50% | Rarely |
|  | Hungary | - | - | - | - | - | - | - | - |
|  | Serbia | - | - | - | - | - | - | - | - |
|  | Georgia | - | - | IgE mediated | Egg, milk, fish, fruit, tree nuts | 3-4 months | >75% | 25-50% | Sometimes |
|  | Latvia | Egg, milk, wheat, fish, carrot (based on clinical practice) | Fish, hazelnut, soy, chicken, potato(based on clinical practice) | Mixed IgE and non-IgE symptoms | Potato, rice, banana, apple, carrot | 4-6 months | 25-50% | <25% | Sometimes |
|  | Belarus | Milk, egg, soy, wheat, fish (based on clinical practice) | Fish, hazelnuts, shrimps, egg, peanut (based on clinical practice) | Non-IgE mediated | Porridge (cereals), vegetable, meat, fruit, egg | 4-6 months | 25-50% | <25% | Sometimes |
|  | Czech R | Cows milk, egg, nuts, wheat, peanut (based on clinical practice) | Fruits, vegetables, nuts, peanuts, poppy (based on clinical practice) | IgE mediated | Carrot, potato, apple, rice, vegetables | 4-6 months | >75% | 25=50% | Often |
|  | Russia | (no data) | - | - | - | - | - | - | - |
|  | Bulgaria | Cows milk. egg soy, kiwi, peach (based on clinical practice) | Peanut, hazelnut, fish, shrimp, vegetables | Non-IgE mediated | Cereals, fruits, vegetables, meats | 4-6 months | >75% | >75% | Sometimes |
|  | Albania | (no data) | - | - | - | - | - | - | - |
|  | Ukraine | (no data) | - | - | - | - | - | - | - |
|  | Moldova | (no data) | - | - | - | - | - | - | - |
|  |  |  |  |  |  |  |  |  |  |
| **Nordic regions** | Denmark | Egg, cows milk, peanut[^50^](#_ENREF_50) (fish and soy) | Vegetables, cows milk, fruits, nuts, legumes, seafood, nuts, wheat [^39^](#_ENREF_39) | IgE mediated | - | - | - | - | - |
|  | Norway | Cows milk, fruits, vegetables, egg, fish, nuts, cereals [^82^](#_ENREF_82) | - | IgE mediated | - | 4-6 months | - | - | Often |
|  | Iceland | Egg, cows milk, fish, wheat, peanuts, soy [^55^](#_ENREF_55) | - | Mixed IgE and non-IgE symptoms | Rice, potato, carrot, apple banana | 4-6 months | <25% | <25% | Sometimes |
|  | Sweden | Cows milk egg, fish, wheat, tree nuts, soy [^56^](#_ENREF_56)^,^ [^83^](#_ENREF_83) | Peanut, tree nut, cows milk, egg, fish, soy [^56^](#_ENREF_56)^,^ [^83^](#_ENREF_83) | IgE mediated | Fruit, carrot, potato, banana, pea | 4-6 months | <25% | 25-50% | Often |
|  | Finland | Cows milk, fruits, vegetales, fish, egg, nuts, wheat [^39^](#_ENREF_39) | Vegetables, nuts, fruits, milk, eggs and grains [^57^](#_ENREF_57) | Mixed IgE and non-IgE symptoms | - | - | - | - | - |

**4) MIDDLE EAST AND AFRICA**

| **Region** | **Country** | **Most common food triggers < 5 years** | | | **Most common symptoms** (IgE, non-IgE, or mixed features) | **First weaning foods** (other than formula milk) | **Age of starting comple-mentary foods** (actual rather than recommended) | **Estimated perinatal use of formulas** (by hospital or nursery staff) | **Estimated use of any formulas**  **< 4 months**  (excluding the neonatal period) | **Is allergy prevention a common parental concern?** |
| --- | --- | --- | --- | --- | --- | --- | --- | --- | --- | --- |
|  |  | **< 5 years** | | **> 5 years** |  |  |  |  |  |  |
|  |  |  | |  |  |  |  |  |  |  |
| **Middle East** | Israel | Milk, egg, sesame, peanut, fish | | Jews: milk, egg, peanut and sesame [^60^](#_ENREF_60)  Arab: Egg, sesame, peanut milk, [^60^](#_ENREF_60) | IgE mediated | Vegetables, fruit, wheat, rice, potato, wheat | 4-6 months | 50-75% | >75% | Sometimes |
|  | United Arab Emirates # | - | | Eggs, fruits, fish, peanuts, tree nuts, cow milk, wheat, vegetables [^61^](#_ENREF_61) | IgE mediated | - | - | - | - | - |
|  | Lebanon | Cows milk, wheat, sesame, tree nuts, peanuts (based on clinical experience) | | Wheat, sesame, shellfish, fish, tree nuts peanuts | IgE mediated | Potato, squash, carrots, rice, wheat | 4-6 months | <25% | 25-50% | Rarely |
|  | Iran | cow's milk, hazelnuts, wheat flour, egg white (12.6%). {Farjadian, | | cow's milk, hazelnuts, wheat flour, egg white (12.6%)[^64^](#_ENREF_64) | IgE mediated | Rice, apple, potato, lamb, carrot | 4-6 months | <25% | 25-50% | Sometimes |
|  | Egypt | Cows milk, egg, sea food, fruits, peanut (based on clinical experience) | | Sea food, egg, fruits, sesame, peanut (based on clinical experience) | IgE mediated | Rice, cereals vegetables, yogurt, egg yolk | 3-4 months | <25% | 25-50% | Sometimes |
|  | Jordan | Cows milk, egg, nuts, peanut, fruit (based on clinical experience) | | Cows milk, egg, nuts, peanuts, vegetables (based on clinical experience) | IgE mediated | - | 3-4 months | <25% | 25-50% | Sometimes |
|  | Kuwait | Cow's milk, eggs, peanuts, wheat, tree nuts (based on clinical experience) | | Peanuts, tree nuts, fish, shell fish (based on clinical | IgE mediated | Rice, cereals, vegetables, fruits, dairy products | 3-4 months | 50-75% | 25-50% | Rarely |
|  | Azerbaijan | (no data) | | - | - | - | - | - | - | - |
|  | Afghanistan | (no data) | | - | - | - | - | - | - | - |
|  | Pakistan | (no data) | | - | - | - | - | - | - | - |
|  |  |  | |  |  |  |  |  | | |
| **Africa** | Ghana # | (no data) | | Peanut, pineapple, pawpaw orange, mango, banana, (SPT) [^65^](#_ENREF_65) | Mixed IgE and non-IgE symptoms | Fruits, vegetables, cereals, meat, egg yolk | - | - | - | - |
|  | Mozambique # | (no data) | | Seafood, meats, fruits and vegetables [^66^](#_ENREF_66) | Mixed IgE and non-IgE symptoms | - | - | - | - | - |
|  | Tanzania # | - | | - | - | - | - | - | - | - |
|  | South Africa | Peanut, egg, milk [^84^](#_ENREF_84) | | Egg, cows milk, peanut soy [^68^](#_ENREF_68) [^69^](#_ENREF_69) | Non-IgE symptoms | Maize, vegetables and fruits | 4-6 months | >75% | >75% | Sometimes |
|  | Morocco | (no data) | 2.5% of children sensitized to food specific IgE (milk, egg, soy, wheat, peanut. Fish) [^70^](#_ENREF_70) | | - | - | - | - | - | - |
|  | Kenya | (no data) | Egg, milk, cabbage, fish (based on specific IgE in adults only) [^72^](#_ENREF_72) | | - | - | - | - | - | - |
|  | Congo# | (no data) | Crab, wheat, soy [^73^](#_ENREF_73) | |  |  |  |  | - |  |
|  | Nigeria | (no data) | Egg, shellfish, milks [^74^](#_ENREF_74) | | - | - | - | - | - | - |
|  | Zimbabwe | (no data) | | Apple, tomato, crab, soy, peanut (based on specific IgE only) [^75^](#_ENREF_75) | - | - | - | - | - | Rarely |
|  | Tunisia | (no data) | | - | - | - | - | - | - | - |
|  | Botswana# |  | | Egg, milk, peanut [^70^](#_ENREF_70) | - | - | - |  |  |  |
|  | Algeria | (no data) | | - | - | - | - | - | - | - |
|  |  |  | |  |  |  |  |  | | |

# Indicates: a country that does *not* have a WAO member society

**Table S3: Food Allergy Health services (in 2012)**

| **Region** | **Country** | **Are there standardized National Anaphylaxis Action Plans?** | **Are adrenaline auto-injectors readily available?** | **Are these subsidized by national health care systems?** | **Are there urban-rural difference in pediatric allergy health care services?** | **Approximate number of pediatric allergists in that country** | **Approximate number of pediatric allergists per million of total population** |
| --- | --- | --- | --- | --- | --- | --- | --- |
|  |  |  |  |  |  |  |  |
| **Oceania** | Australia | Yes | Yes | Yes | Yes | 70 | 3.0 |
|  | New Zealand | Yes | Yes | No | Yes | 2 | 0.45 |
|  |  |  |  |  |  |  |  |
| **Asia** | China | No | No | - | Yes | - | - |
|  | Thailand | No | Yes | Yes | Yes | 120 | 1.87 |
|  | Taiwan | - | - | - | - | - | - |
|  | Japan | Yes | Yes | Yes | Yes | 937 | 7.34 |
|  | Korea | Yes | Yes | Yes | Yes | 133 | 2.77 |
|  | Hong Kong | - | - | - | No | - | - |
|  | Singapore | Yes | Yes | No | No | 10* | 2.00 |
|  | Philippines | No | No | - | Yes | 67 | 0.69 |
|  | Indonesia | No | No |  | Yes | 38 | 0.16 |
|  | Malaysia | No | No | - | Yes | 4 | 0.14 |
|  | Burma | - | - | - | Yes | - | - |
|  | Bangladesh | No | No | - | Yes | - | - |
|  | Sri Lanka | - | - | - | Yes | - | - |
|  | Vietnam | - | - | - | Yes | - | - |
|  | India | No | No | - | Yes | 10* | 0.008 |
|  | Mongolia | - | - |  | No | 6 | 2.14 |
|  |  |  |  |  |  |  |  |
| **North America** | Canada | No | Yes | Yes | Yes | 75 | 2.14 |
|  | USA | Yes | Yes | Yes | Yes | 2591 | 8.28 |
|  |  |  |  |  |  |  |  |
| **South America** | Colombia | - | - | - | - | - | - |
|  | Mexico | No | No | - | No | 254 | 2.35 |
|  | Panama | No | No | - | Yes | 7 | 2.19 |
|  | Honduras | No | No | - | Yes | 14 | 2.33 |
|  | Argentina | No | Yes | No | Yes | - | - |
|  | Uruguay | No | No | - | No | 6* | 1.8 |
|  | Brazil | No | Yes | No | Yes | 0 | - |
|  | Chile | No | Yes | No | Yes | 50* | 3.0 |
|  | Cuba | Yes | No | - | No | 110 | 9.7 |
|  | Peru | - | - | - | Yes | - | - |
|  | Venezuela | - | - | - | Yes | - | - |
|  | Ecuador | - | - | - | Yes | - | - |
|  | Paraguay | - | - | - | - | - | - |
|  |  |  |  |  |  |  |  |
| **Western Europe** | UK | No | Yes | Yes | Yes | 20 | 0.32 |
|  | Germany | Yes | Yes | Yes | Yes | 886 | 10.84 |
|  | Switzerland | Yes | Yes | Yes | Yes | 29 | 3.6 |
|  | Greece | Yes | Yes | Yes | Yes | 110 | 10.2 |
|  | Poland | - | - | - | No | - | - |
|  | Netherlands | No | Yes | Yes | No | 6 | 0.38 |
|  | Belgium | No | Yes | Yes | No | - | - |
|  | France | - | - | - | - | - | - |
|  | Austria | No | Yes | Yes | No | - | - |
|  | Spain | Yes | Yes | Yes | No | - | - |
|  | Italy | - | - | - | Yes | 450 | 7.5 |
|  | Portugal | No | Yes | Yes | Yes | - | - |
|  |  |  |  |  |  |  | - |
| **Central/**  **Eastern Europe** | Turkey | Yes | Yes | Yes | Yes | 153 | 2.02 |
|  | Lithuania | - | - | - | Yes | - | - |
|  | Slovenia | - | - | - | Yes | - | - |
|  | Estonia | - | - |  | Yes | - | - |
|  | Croatia | Yes | Yes | Yes | Yes | 15 | 3.49 |
|  | Romania | No | No | - | Yes | 30 | 1.55 |
|  | Hungary | - | - | - | Yes | - | - |
|  | Serbia | - | - | - | - | - | - |
|  | Georgia | Yes | Yes | No | Yes | 72 | 14.4 |
|  | Latvia | No | Yes | No | Yes | 12 | 6.0 |
|  | Belarus | Yes | No | - | Yes | 20 | 2.11 |
|  | Czech R | Yes | Yes | Yes | No | 100* | 9.52 |
|  | Russia | - | - | - | - | - | - |
|  | Bulgaria | - | - | - | Yes | 34 | 4.62 |
|  | Albania | - | - | - | - | - | - |
|  | Ukraine | - | - | - | - | - | - |
|  | Moldova | - | - | - | - | - | - |
|  |  |  |  |  |  |  |  |
| Nordic regions | Denmark | Yes | Yes | Yes | Yes | - | - |
|  | Norway | Yes | Yes | Yes | Yes | - | - |
|  | Iceland | No | Yes | Yes | Yes | 8 | 25.0 |
|  | Sweden | Yes | Yes | Yes | No | 80 | 8.40 |
|  | Finland | Yes | Yes | Yes | Yes | - | - |
|  |  |  |  |  |  |  |  |
| Middle East | Israel | Yes | Yes | Yes | No | 70 | 8.75 |
|  | United Arab Emirates # | - | - | - | - | - | - |
|  | Lebanon | No | No | - | No | 4 | 1.00 |
|  | Iran | - | - | - | - | 56 | 0.74 |
|  | Egypt | No | No | - | Yes | 20 | 0.24 |
|  | Jordan | Yes | No | - | No | 8 | 1.33 |
|  | Kuwait |  |  |  |  | 5 | 1.77 |
|  | Azerbaijan | - | - | - | - | - | - |
|  | Afghanistan | - | - | - | - | - | - |
|  | Pakistan | - | - | - | - | - | - |
|  |  |  |  |  |  |  | - |
| Africa | Ghana # | - | - | - | - | - | - |
|  | Mosam-bique # | - | - | - | - | - | - |
|  | Tanzania # | - | - | - | - | - | - |
|  | South Africa | No | No | - | Yes | 8 | 0.57 |
|  | Morocco | - | - | - | - | - | - |
|  | Kenya | - | - | - | - | - | - |
|  | Congo# | - | - | - | - | - | - |
|  | Nigeria # | - | - | - | - | - | - |
|  | Zimbabwe | - | - | - | Yes | 0 | 0 |
|  | Tunisia | - | - | - | - | - | - |
|  | Botswana# | - | - | - | - | - | - |
|  | Algeria | - | - | - | - | - | - |
|  |  |  |  |  |  |  |  |

**Table References:**

1. Osborne NJ, Koplin JJ, Martin PE, Gurrin LC, Lowe AJ, Matheson MC, et al. Prevalence of challenge-proven IgE-mediated food allergy using population-based sampling and predetermined challenge criteria in infants. J Allergy Clin Immunol 2011; 127:668-76 e1-2.

2. Kljakovic M, Gatenby P, Hawkins C, Attewell RG, Ciszek K, Kratochvil G, et al. The parent-reported prevalence and management of peanut and nut allergy in school children in the Australian Capital Territory. J Paediatr Child Health 2009; 45:98-103.

3. Poulos LM, Waters AM, Correll PK, Loblay RH, Marks GB. Trends in hospitalizations for anaphylaxis, angioedema, and urticaria in Australia, 1993-1994 to 2004-2005. J Allergy Clin Immunol 2007; 120:878-84.

4. Mullins RJ. Paediatric food allergy trends in a community-based specialist allergy practice, 1995-2006. Med J Aust 2007; 186:618-21.

5. Liew WK, Williamson E, Tang ML. Anaphylaxis fatalities and admissions in Australia. J Allergy Clin Immunol 2009; 123:434-42.

6. Crooks C, Ameratunga R, Simmons G, Jorgensen P, Wall C, Brewerton M, et al. The changing epidemiology of food allergy--implications for New Zealand. N Z Med J 2008; 121:74-82.

7. Leung TF, Yung E, Wong YS, Lam CW, Wong GW. Parent-reported adverse food reactions in Hong Kong Chinese pre-schoolers: epidemiology, clinical spectrum and risk factors. Pediatr Allergy Immunol 2009; 20:339-46.

8. Chen J, Hu Y, Allen KJ, Ho MHK, Li H. The Prevalence of Food Allergy in Infants in Chongqing, China. Paediatric Allergy Immunology. 2011; 22(4):356-60.

9. Chen J, Liao Y, Zhang HZ, Zhao H, Chen J, Li HQ. Prevalence of food allergy in children under 2 years of age in three cities in China. Zhonghua Er Ke Za Zhi 2012; 50:5-9.

10. Hu Y, Chen J, Li H. Comparison of food allergy prevalence among Chinese infants in Chongqing, 2009 versus 1999. Pediatr Int 2010; 52:820-4.

11. Lao-araya M, Trakultivakorn M. Prevalence of food allergy among preschool children in northern Thailand. Pediatr Int 2012; 54:238-43.

12. Santadusit S, Atthapaisalsarudee S, Vichyanond P. Prevalence of adverse food reactions and food allergy among Thai children. J Med Assoc Thai 2005; 88 Suppl 8:S27-32.

13. Wu TC, Tsai TC, Huang CF, Chang FY, Lin CC, Huang IF, et al. Prevalence of food allergy in Taiwan: a questionnaire-based survey. Intern Med J 2012; 42:1310-5.

14. Kim J, Chang E, Han Y, Ahn K, Lee SI. The incidence and risk factors of immediate type food allergy during the first year of life in Korean infants: a birth cohort study. Pediatr Allergy Immunol 2011; 22:715-9.

15. Ahn K, Kim J, Hahm MI, Lee SY, Kim WK, Chae Y, et al. Prevalence of immediate-type food allergy in Korean schoolchildren: A population-based study. Allergy Asthma Proc 2012; 33:481-7.

16. Oh J, Pyun B, Choung J, Ahn K, Kim C, Song S, et al. Epidemiological Change of Atopic Dermatitis and Food Allergy in School-Aged Children in Korea between 1995 and 2000. J Korean Med Sci. 2004; 19(5):716-23.

17. Noda R. Prevalence of food allergy in nursery school (nationwide survey). Jpn J Food Allergy 2010:5-9.

18. Kusunoki T, Morimoto T, Nishikomori R, Heike T, Fujii T, Nakahata T. Allergic status of schoolchildren with food allergy to eggs, milk or wheat in infancy. Pediatr Allergy Immunol 2009; 20:642-7.

19. Kusunoki T, Morimoto T, Sakuma M, Mukaida K, Yasumi T, Nishikomori R, et al. Effect of eczema on the association between season of birth and food allergy in Japanese children. Pediatr Int 2013; 55:7-10.

20. Lee AJ, Thalayasingam M, Lee BW. Food allergy in Asia: how does it compare? Asia Pac Allergy 2013; 3:3-14.

21. Ho MH, Lee SL, Wong WH, Ip P, Lau YL. Prevalence of self-reported food allergy in Hong Kong children and teens--a population survey. Asian Pac J Allergy Immunol 2012; 30:275-84.

22. Shek LP, Cabrera-Morales EA, Soh SE, Gerez I, Ng PZ, Yi FC, et al. A population-based questionnaire survey on the prevalence of peanut, tree nut, and shellfish allergy in 2 Asian populations. J Allergy Clin Immunol 2010; 126:324-31, 31 e1-7.

23. Connett GJ, Gerez I, Cabrera-Morales EA, Yuenyongviwat A, Ngamphaiboon J, Chatchatee P, et al. A population-based study of fish allergy in the Philippines, Singapore and Thailand. Int Arch Allergy Immunol 2012; 159:384-90.

24. Soller L, Ben-Shoshan M, Harrington DW, Fragapane J, Joseph L, St Pierre Y, et al. Overall prevalence of self-reported food allergy in Canada. J Allergy Clin Immunol 2012; 130:986-8.

25. Ben-Shoshan M, Turnbull E, Clarke A. Food allergy: temporal trends and determinants. Curr Allergy Asthma Rep 2012; 12:346-72.

26. Ben-Shoshan M, Kagan RS, Alizadehfar R, Joseph L, Turnbull E, St Pierre Y, et al. Is the prevalence of peanut allergy increasing? A 5-year follow-up study in children in Montreal. J Allergy Clin Immunol 2009; 123:783-8.

27. Taylor-Black S, Wang J. The prevalence and characteristics of food allergy in urban minority children. Ann Allergy Asthma Immunol 2012; 109:431-7.

28. Gupta RS, Springston EE, Warrier MR, Smith B, Kumar R, Pongracic J, et al. The prevalence, severity, and distribution of childhood food allergy in the United States. Pediatrics 2011; 128:e9-17.

29. Branum AM, Lukacs SL. Food allergy among children in the United States. Pediatrics 2009; 124:1549-55.

30. Ross MP, Ferguson M, Street D, Klontz K, Schroeder T, Luccioli S. Analysis of food-allergic and anaphylactic events in the National Electronic Injury Surveillance System. J Allergy Clin Immunol 2008; 121:166-71.

31. Marrugo J, Hernandez L, Villalba V. Prevalence of self-reported food allergy in Cartagena (Colombia) population. Allergol Immunopathol (Madr) 2008; 36:320-4.

32. Rodriguez-Ortiz PG, Munoz-Mendoza D, Arias-Cruz A, Gonzalez-Diaz SN, Herrera-Castro D, Vidaurri-Ojeda AC. Epidemiological characteristics of patients with food allergy assisted at Regional Center of Allergies and Clinical Immunology of Monterrey. Rev Alerg Mex 2009; 56:185-91.

33. McBride D, Keil T, Grabenhenrich L, Dubakiene R, Drasutiene G, Fiocchi A, et al. The EuroPrevall birth cohort study on food allergy: baseline characteristics of 12,000 newborns and their families from nine European countries. Pediatr Allergy Immunol 2012; 23:230-9.

34. Venter C, Pereira B, Grundy J, Clayton CB, Roberts G, Higgins B, et al. Incidence of parentally reported and clinically diagnosed food hypersensitivity in the first year of life. J Allergy Clin Immunol 2006; 117:1118-24.

35. Venter C, Pereira B, Grundy J, Clayton CB, Arshad SH, Dean T. Prevalence of sensitization reported and objectively assessed food hypersensitivity amongst six-year-old children: a population-based study. Pediatr Allergy Immunol 2006; 17:356-63.

36. Venter C, Pereira B, Voigt K, Grundy J, Clayton CB, Higgins B, et al. Prevalence and cumulative incidence of food hypersensitivity in the first 3 years of life. Allergy 2008; 63:354-9.

37. Venter C, Hasan Arshad S, Grundy J, Pereira B, Bernie Clayton C, Voigt K, et al. Time trends in the prevalence of peanut allergy: three cohorts of children from the same geographical location in the UK. Allergy 2010; 65:103-8.

38. Roehr CC, Edenharter G, Reimann S, Ehlers I, Worm M, Zuberbier T, et al. Food allergy and non-allergic food hypersensitivity in children and adolescents. Clin Exp Allergy 2004; 34:1534-41.

39. Steinke M, Fiocchi A, Kirchlechner V, Ballmer-Weber B, Brockow K, Hischenhuber C, et al. Perceived food allergy in children in 10 European nations. A randomised telephone survey. Int Arch Allergy Immunol 2007; 143:290-5.

40. Brugman E, Meulmeester JF, Spee-van der Wekke A, Beuker RJ, Radder JJ, Verloove-Vanhorick SP. Prevalence of self-reported food hypersensitivity among school children in The Netherlands. Eur J Clin Nutr 1998; 52:577-81.

41. Rance F, Grandmottet X, Grandjean H. Prevalence and main characteristics of schoolchildren diagnosed with food allergies in France. Clin Exp Allergy 2005; 35:167-72.

42. Kanny G, Moneret-Vautrin DA, Flabbee J, Beaudouin E, Morisset M, Thevenin F. Population study of food allergy in France. J Allergy Clin Immunol 2001; 108:133-40.

43. Fernandez Rivas M. Food allergy in Alergologica-2005. J Investig Allergol Clin Immunol 2009; 19 Suppl 2:37-44.

44. Caffarelli C, Coscia A, Ridolo E, Povesi Dascola C, Gelmett C, Raggi V, et al. Parents' estimate of food allergy prevalence and management in Italian school-aged children. Pediatr Int 2011; 53:505-10.

45. Mustafayev R, Civelek E, Orhan F, Yuksel H, Boz AB, Sekerel BE. Similar prevalence, different spectrum: IgE-mediated food allergy among Turkish adolescents. Allergol Immunopathol (Madr) 2012.

46. Orhan F, Karakas T, Cakir M, Aksoy A, Baki A, Gedik Y. Prevalence of immunoglobulin E-mediated food allergy in 6-9-year-old urban schoolchildren in the eastern Black Sea region of Turkey. Clin Exp Allergy 2009; 39:1027-35.

47. Dubakiene R, Surkiene G, Stukas R, Pirmaityte-Vilesko J, Kavaliunas A. Food allergies among 5th-9th grade school- children in Vilnius (Lithuania). Ekologija 2008; 54(1):1-4.

48. Kavaliunas A, Surkiene G, Dubakiene R, Stukas R, Zagminas K, Saulyte J, et al. EuroPrevall survey on prevalence and pattern of self-reported adverse reactions to food and food allergies among primary schoolchildren in Vilnius, Lithuania. Medicina (Kaunas) 2012; 48:265-71.

49. Sandin A, Annus T, Bjorksten B, Nilsson L, Riikjarv MA, van Hage-Hamsten M, et al. Prevalence of self-reported food allergy and IgE antibodies to food allergens in Swedish and Estonian schoolchildren. Eur J Clin Nutr 2005; 59:399-403.

50. Eller E, Kjaer HF, Host A, Andersen KE, Bindslev-Jensen C. Food allergy and food sensitization in early childhood: results from the DARC cohort. Allergy 2009; 64:1023-9.

51. Osterballe M, Hansen TK, Mortz CG, Host A, Bindslev-Jensen C. The prevalence of food hypersensitivity in an unselected population of children and adults. Pediatr Allergy Immunol 2005; 16:567-73.

52. Kjaer HF, Eller E, Host A, Andersen KE, Bindslev-Jensen C. The prevalence of allergic diseases in an unselected group of 6-year-old children. The DARC birth cohort study. Pediatr Allergy Immunol 2008; 19:737-45.

53. Kvenshagen B, Halvorsen R, Jacobsen M. Is there an increased frequency of food allergy in children delivered by caesarean section compared to those delivered vaginally? Acta Paediatr 2009; 98:324-7.

54. Eggesbo M, Botten G, Stigum H, Nafstad P, Magnus P. Is delivery by cesarean section a risk factor for food allergy? J Allergy Clin Immunol 2003; 112:420-6.

55. Kristinsdóttir H, Clausen M, Ragnarsdóttir HS, Halldórsdóttir IH, McBride D, Beyer K, et al. Prevalence of food allergy in Icelandic infants during first year of life. Laeknabladid (Article in Icelandic) 2011; 97(1):11-8.

56. Ostblom E, Lilja G, Pershagen G, van Hage M, Wickman M. Phenotypes of food hypersensitivity and development of allergic diseases during the first 8 years of life. Clin Exp Allergy 2008; 38:1325-32.

57. Kallio P, Salmivesi S, Kainulainen H, Paassilta M, Korppi M. Parent-reported food allergy requiring an avoidance diet in children starting elementary school. Acta Paediatr 2011; 100:1350-3.

58. Kajosaari M. Food allergy in Finnish children aged 1 to 6 years. Acta Paediatr Scand 1982; 71:815-9.

59. Pyrhonen K, Nayha S, Kaila M, Hiltunen L, Laara E. Occurrence of parent-reported food hypersensitivities and food allergies among children aged 1-4 yr. Pediatr Allergy Immunol 2009; 20:328-38.

60. Graif Y, German L, Livne I, Shohat T. Association of food allergy with asthma severity and atopic diseases in Jewish and Arab adolescents. Acta Paediatr 2012; 101:1083-8.

61. Al-Hammadi S, Al-Maskari F, Bernsen R. Prevalence of food allergy among children in Al-Ain city, United Arab Emirates. Int Arch Allergy Immunol 2010; 151:336-42.

62. Irani C, Maalouly G, Germanos M, Kazma H. Food allergy in Lebanon: is sesame seed the "middle eastern" peanut. World Allergy Organ J 2011; 4:1-3.

63. Moghtaderi M, Farjadian S, Kashef S, Tavakoli M, Alyasin S, Afrasiab M, et al. Specific IgE to Common Food Allergens in Children with Atopic Dermatitis. Iran J Immunol 2012; 9:32-8.

64. Farjadian S, Moghtaderi M, Kashef S, Alyasin S. Sensitization to food allergens in Iranian children with mild to moderate persistent asthma. World J Pediatr 2012; 8:317-20.

65. Obeng BB, Amoah AS, Larbi IA, Yazdanbakhsh M, van Ree R, Boakye DA, et al. Food allergy in Ghanaian schoolchildren: data on sensitization and reported food allergy. Int Arch Allergy Immunol 2011; 155:63-73.

66. Lunet N, Falcao H, Sousa M, Bay N, Barros H. Self-reported food and drug allergy in Maputo, Mozambique. Public Health 2005; 119:587-9.

67. Justin-Temu M, Risha P, Abla O, Massawe A. Incidence, knowledge and health seeking behaviour for perceived allergies at household level: a case study in Ilala district Dar es Salaam Tanzania. East Afr J Public Health 2008; 5:90-3.

68. Levin ME, Le Souef PN, Motala C. Total IgE in urban Black South African teenagers: the influence of atopy and helminth infection. Pediatr Allergy Immunol 2008; 19:449-54.

69. Potter PC, de Longueville M. Sensitisation to aero-allergens and food allergens in infants with atopic dermatitis in South Africa. . Curr Allergy Clin Immunol 2005; 18 (3):131-2.

70. Kung SJ, Steenhoff AP, Gray C. Food Allergy in Africa: Myth or Reality? Clin Rev Allergy Immunol 2012.

71. De Souza M. Allergic conditions in a general practice in Nairobi: a pilot study. East African Medical Journal 1992; 69:700-2.

72. De Souza M. Allergies and skin testing: a Nairobi experience. East African Medical Journa 1994; 171:473-5.

73. Nyembue TD, Ntumba W, Omadjela LA, Muyunga C, Hellings PW, Jorissen M. Sensitization rate and clinical profile of Congolese patients with rhinitis. Allergy Rhinol (Providence) 2012; 3:e16-24.

74. Nnoruka EN. Current epidemiology of atopic dermatitis in south-eastern Nigeria. Int J Dermatol 2004; 43:739-44.

75. Westritschnig K, Sibanda E, Thomas W, Auer H, Aspock H, Pittner G, et al. Analysis of the sensitization profile towards allergens in central Africa. Clin Exp Allergy 2003; 33:22-7.

76. Schnabel E, Sausenthaler S, Schaaf B, Schafer T, Lehmann I, Behrendt H, et al. Prospective association between food sensitization and food allergy: results of the LISA birth cohort study. Clin Exp Allergy 2010; 40:450-7.

77. Zutavern A, Brockow I, Schaaf B, Bolte G, von Berg A, Diez U, et al. Timing of solid food introduction in relation to atopic dermatitis and atopic sensitization: results from a prospective birth cohort study. Pediatrics 2006; 117:401-11.

78. Ferrari GG, Eng PA. IgE-mediated food allergies in Swiss infants and children. Swiss Med Wkly 2011; 141:w13269.

79. Karakoc G, Keskin O, Asilsoy S, al. e. Food Allergy profile in tertiary clinics in SE part of Turkey. Poster presentations in Turkish National Society of Allergy and Clinical Immunology Annual Congress 2012.

80. Yavuz ST, Sahiner UM, Buyuktiryaki B, al e. Phenotypes of IgE-mediated food allergy in Turkish children. Allergy Asthma Proc 2011; 32(6) 47-55.

81. Dubakiene R, Rudzeviciene O, Butiene I, Sezaite I, Petronyte M, Vaicekauskaite D, et al. Studies on early allergic sensitization in the Lithuanian birth cohort. ScientificWorldJournal 2012; 2012:909524.

82. Eggesbo M, Halvorsen R, Tambs K, Botten G. Prevalence of parentally perceived adverse reactions to food in young children. Pediatr Allergy Immunol 1999; 10:122-32.

83. Ostblom E, Wickman M, van Hage M, Lilja G. Reported symptoms of food hypersensitivity and sensitization to common

foods in 4-year-old children. Acta Pædiatrica 2008; 97: 85–90.

84. Gray C, Kung SJ. Food allergy in south africa: joining the food allergy epidemic? Curr Allergy Clin Immunol 2012; 25:25-9.
